# Statistical batch-aware embedded integration, dimension reduction, and alignment for spatial transcriptomics

**DOI:** 10.1093/bioinformatics/btae611

**Published:** 2024-10-14

**Authors:** Yanfang Li, Shihua Zhang

**Affiliations:** NCMIS, CEMS, RCSDS, Academy of Mathematics and Systems Science, Chinese Academy of Sciences, Beijing 100190, China; NCMIS, CEMS, RCSDS, Academy of Mathematics and Systems Science, Chinese Academy of Sciences, Beijing 100190, China; School of Mathematical Sciences, University of Chinese Academy of Sciences, Beijing 100049, China; Key Laboratory of Systems Health Science of Zhejiang Province, School of Life Science, Hangzhou Institute for Advanced Study, University of Chinese Academy of Sciences, Hangzhou 310024, China

## Abstract

**Motivation:**

Spatial transcriptomics (ST) technologies provide richer insights into the molecular characteristics of cells by simultaneously measuring gene expression profiles and their relative locations. However, each slice can only contain limited biological variation, and since there are almost always non-negligible batch effects across different slices, integrating numerous slices to account for batch effects and locations is not straightforward. Performing multi-slice integration, dimensionality reduction, and other downstream analyses separately often results in suboptimal embeddings for technical artifacts and biological variations. Joint modeling integrating these steps can enhance our understanding of the complex interplay between technical artifacts and biological signals, leading to more accurate and insightful results.

**Results:**

In this context, we propose a hierarchical hidden Markov random field model STADIA to reduce batch effects, extract common biological patterns across multiple ST slices, and simultaneously identify spatial domains. We demonstrate the effectiveness of STADIA using five datasets from different species (human and mouse), various organs (brain, skin, and liver), and diverse platforms (10x Visium, ST, and Slice-seqV2). STADIA can capture common tissue structures across multiple slices and preserve slice-specific biological signals. In addition, STADIA outperforms the other three competing methods (PRECAST, fastMNN, and Harmony) in terms of the balance between batch mixing and spatial domain identification, and it demonstrates the advantage of joint modeling when compared to STAGATE and GraphST.

**Availability and implementation:**

The source code implemented by R is available at https://github.com/zhanglabtools/STADIA and archived with version 1.01 on Zenodo https://zenodo.org/records/13637744.

## 1 Introduction

Spatial transcriptomics (ST) technologies can measure gene expression profiles and their relative spatial locations, providing new opportunities and challenges for computational biologists. Various methods have been developed to analyze ST data, including spatial domain identification (Fu *et al.* 2021, Zhao *et al.* 2021, Hu *et al.* 2021a, Dong and Zhang 2022), spatially variable genes (SVGs) detection (Edsgärd *et al.* 2018, Svensson *et al.* 2018, Sun *et al.* 2020, Andersson and Lundeberg 2021, Zhu *et al.* 2021, Zhang *et al.* 2023), and spatially aware cell type deconvolution (Elosua-Bayes *et al.* 2021, Shan *et al.* 2022, Ma and Zhou 2022, Lu *et al.* 2023), and so on. However, most of these approaches only focus on individual ST slices, which may limit their utility for multi-slice analysis. Nevertheless, multi-slice integrative analysis is fundamental for the comprehensive exploration of ST data. It reveals hidden patterns and relationships that may remain concealed when focusing solely on individual slices and enables researchers to capture the intricate spatiotemporal dynamics of gene expression across different slices, providing a more holistic perspective of the underlying biological process. Therefore, it is crucial to consider the integration of multiple ST slices by modeling the gene expression and spatial locations carefully.

When merging multiple slices collected under different conditions, laboratories, or experimenters, there are more or less unwanted factors, often referred to as batch effects. If left unadjusted, these batch effects can mask the biological variation of interest, potentially leading to inaccurate results in downstream analysis. To address this issue, researchers have developed several batch effect correction strategies for single-cell RNA sequencing (scRNA-seq), including ComBat (Johnson *et al.* 2007), Harmony (Korsunsky *et al.* 2019), fastMNN (Haghverdi *et al.* 2018), Scanorama (Hie *et al.* 2019), and Seurat-CCA (Hardoon *et al.* 2004, Stuart *et al.* 2019). However, these approaches are primarily tailored for the removal of batch effects in scRNA-seq without considering the relative locations of different cells. Therefore, applying these methods to multiple ST datasets may not yield the desired results.

In addition, batch effect correction and downstream analysis are usually performed separately both for scRNA-seq (Hardoon *et al.* 2004, Johnson *et al.* 2007, Haghverdi *et al.* 2018, Korsunsky *et al.* 2019, Hie *et al.* 2019, Stuart *et al.* 2019) and ST (Zeira *et al.* 2022, Long *et al.* 2023, Zhou *et al.* 2023), which may lead to suboptimal results, as it may not effectively account for the interplay between technical variability and biological variations. This limitation is evident, except for several recent approaches such as BUS (Luo and Wei 2019), BFR.BE (Avalos-Pacheco *et al.* 2022), and PRECAST (Liu *et al.* 2023). BUS corrects batch effects and discovers subtypes by integrating the location-and-scale (L/S) adjustment model (Johnson *et al.* 2007) with the Gaussian mixture model (GMM) (Fraley and Raftery 2002, McLachlan *et al.* 2019) for the analysis of microarray data. BFR.BE is a general sparse factor regression model designed for dimension reduction and batch effect correction. Note that neither BUS nor BFR.BE takes into account the relative positions of different cells. PRECAST jointly estimates low-dimensional representations of biological and systematic variation by factor analysis, while simultaneously conducting spatial clustering using GMM with a latent Markov random field Potts (Graner and Glazier 1992) model. However, PRECAST makes certain assumptions, namely (1) that non-cellular biological variation or batch effects are not orthogonal to the biological space, and the projection of batch effects onto the orthogonal complement of the biological space is discarded, and (2) that local neighboring microenvironments are spatially correlated, captured by an intrinsic conditional autoregressive (CAR) model.

To this end, we develop a new ST Analysis tool for multi-slice integration, DImension reduction and Alignment (STADIA). STADIA is a hierarchical hidden Markov random field model, adapting the BUS and BFR.BE algorithms by further accounting for the relative physical positions of different spots/beads, and relaxing the above two assumptions of PRECAST. Extensive experiments conducted on five ST datasets, selected to comprehensively account for factors such as spatial resolution, spatial coherence, source species, and disease status (see Supplementary Table S4 for details), along with comparisons with three competing methods (PRECAST, fastMNN, and Harmony) demonstrate the superior performance of STADIA. Notably, STADIA simultaneously corrects batch effects identifies shared and slice-specific spatial domains across multiple ST slices, and provide insights into the interpretable different variations among distinct slices, including both additive and multiplicative differences, all within a unified framework. To further validate this, we compared STADIA with two deep learning-based methods, STAGATE (Dong and Zhang 2022) and GraphST (Long *et al.* 2023), using two adjacent sagittal mouse brain slices (10x Visium).

## 2 Materials and methods

### 2.1 Overview of STADIA

**Figure 1.**
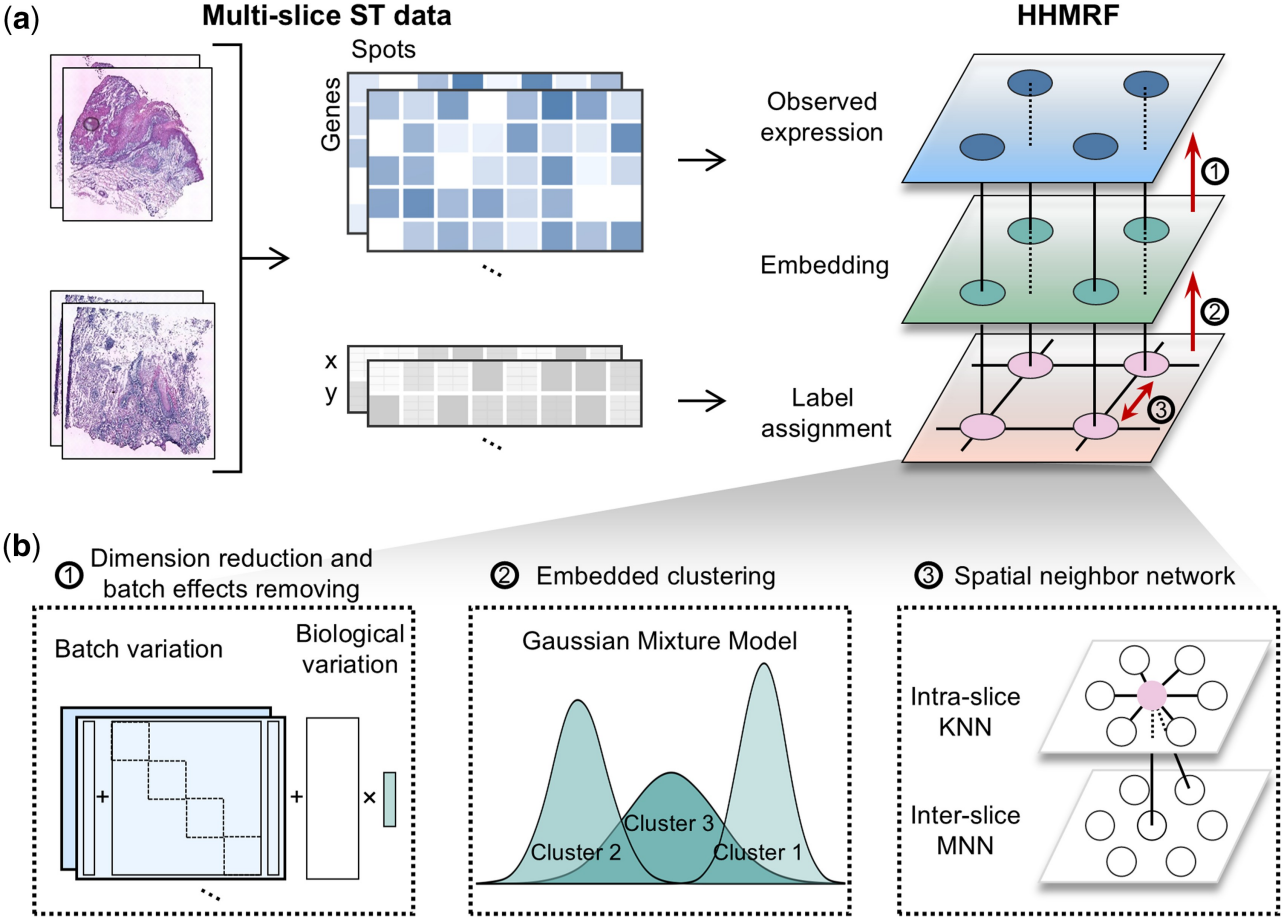
Overview of STADIA. (a) STADIA is a hierarchical hidden Markov random field model (HHMRF) with multi-slice data as input. (b) After normal preprocessing on gene expression profiles, STADIA first uses factor analysis and location-and-scale (L/S) adjustment to perform linear dimension reduction and batch effect correction. Then, STADIA uses the Gaussian mixture model to do embedded clustering. Finally, to ensure that the label assignments are locally consistent, STADIA adopts the Markov random field Potts model on the graph, with nodes being spots of all samples and edges being K-nearest neighbors (KNN) pairs using coordinates within the batch and mutual nearest neighbors (MNN) pairs using gene expression profiles across batches.

STADIA takes the pre-processed gene expression profiles and their corresponding spatial coordinates of multiple ST slices as input. STADIA is a hierarchical hidden Markov random field model (HHMRF) consisting of two hidden states: low-dimensional batch-corrected embeddings and spatially aware cluster assignments (Fig. 1a). Specifically, STADIA first performs both linear dimension reduction and batch effect correction using a Bayesian factor regression model with L/S adjustment. Then, STADIA uses the GMM for embedded clustering. Finally, to ensure local consistency of label assignments, STADIA applies the Potts model on an undirected graph, where nodes are spots from all slices and edges are intra-batch KNN pairs using coordinates and inter-batch MNN pairs using gene expression profiles (Fig. 1b). STADIA utilizes the expectation-maximization (EM) algorithm for parameter estimation, which iteratively estimates the missing or latent variables from the observed data in the E-step and then updates the parameters to maximize the posterior distribution.

### 2.2 Construction of the spatial neighborhood graph

We constructed a combined spatial neighborhood graph consisting of all spots from all slices. Two spots within a slice were connected by an edge if the distance between them was among the K-th (K=4 for ST spots and K=6 for Visium spots) smallest Euclidean distances or less than a predefined radius *r* (r=50 for slide-seqV2) (see subsection “Principal to build the spatial neighborhood graph” in Supplementary Material for general principals). Two spots between any two ST slices were connected if they are mutual nearest neighbors (MNN) (K=2) based on their gene expression profiles. The undirected graph was represented by an adjacency matrix ***A***, where $A_{ij}=1$ if and only if there is an edge between spots *i* and *j*.

### 2.3 The overall architecture of STADIA

Suppose there are *B* batches. Let $y_{bi}\in R^{p}$ be the observed preprocessed gene expression profile for sample *i* in batch *b* ($i=1,\ldots ,n_{b}$), where *p* is the number of genes measured. Further, denote the true expression levels for sample *i* in batch *b* by $x_{bi}\in R^{p}$ and the vector of batch effects by $\gamma_{b}\in R^{p}$. Then inspired by the L/S adjustment modeling to remove batch effects for multi-source scRNA-seq (Johnson *et al.* 2007), gene expression profiles $y_{bi}$ from different batches or slices could be formulated as

(1) $$y_{bi}=x_{bi}+\gamma_{b}+\varepsilon_{bi},$$

where $\varepsilon_{bi}\in R^{p}$ is Gaussian noise with batch-specific diagonal precision matrix $T_{b}$ that has a distribution equal to $T_{b}^{-1/2}N_{p}(0,I)$, where $N_{p}(\cdot )$ denotes *p*-dimensional Gaussian distribution. The vectors $\gamma_{b}$ and $\varepsilon_{bi}$ correct for shifts and proportional changes induced by factors such as variations in instrument calibration and instrument sensitivity, which are referred to as additive and multiplicative batch effects, respectively.

Moreover, the number of genes, *p*, is always ultra-high nowadays due to high-throughput sequencing technology, which is often assumed to lie on a smooth low-dimensional manifold $S^{d}$ (d≪p). Use the linear dimension reduction technique, factor analysis, on $x_{bi}$

(2) $$x_{bi}=Lf_{bi},$$

where $f_{bi}\in R^{d}$ is the common factor score for sample *i* in batch *b* and $L\in R^{p\times d}$ is the loading matrix shared by all batches. Putting (1) and (2) together, we get the first layer of STADIA

(3) $$y_{bi}=Lf_{bi}+\gamma_{b}+\varepsilon_{bi},\;\text{with}\;\varepsilon_{bi}∼N_{p}(0,T_{b}^{-1}).$$

Furthermore, assuming that all samples come from *q* latent clusters, with cluster indicator $c_{bi}$ for sample *i* in batch *b* and given $c_{bi}=k$(k∈[1,…,q]), the latent low-dimensional representation $f_{bi}$ is normally distributed. More specifically, the second layer of STADIA, which connects the hidden batch-corrected low-dimensional representation and the latent cluster assignments, is a GMM

(4) $$f_{bi}|c_{bi}=k,\omega_{bi}∼N_{d}\left(\mu_{k},\omega_{bi}^{-1}\Lambda^{-1}\right),$$

where $\mu_{k}\in R^{d}$ is the mean gene expression for the *k*th cluster, $\omega_{bi}\in R$ and $\Lambda \in R^{d\times d}$ are sample-specific and common precision, respectively. Note that in such a setting, if the prior distribution of $\omega_{bi}$ is given by the gamma distribution $G(\nu_{\omega }/2,\nu_{\omega }/2)$, the marginal distribution of $f_{bi}$ by integrating out $\omega_{bi}$ is the multivariate Student-*t* distribution $t_{d}\left(\nu_{\omega },\mu_{k},\Lambda \right)$ with $\nu_{\omega }$ degrees of freedom (Liu and Rubin 1995), which is more robust to noise and outliers as specified in BayesSpace (Zhao *et al.* 2021).

Finally, since samples close in a physical location within a batch and samples with similar expression across batches tend to have the same biological variations, we use KNN using spatial locations within the slice and MNN using gene expression profiles across slices to construct an undirected graph G=(V,E) with nodes V representing all spots from all batches. Based on the graph G, the cluster indicator c is modeled by the Potts model (Graner and Glazier 1992),

(5) $$P(c)=\prod_{b=1}^{B}\prod_{i=1}^{n_{b}}P(c_{bi})=\prod_{b=1}^{B}\prod_{i=1}^{n_{b}}C(\eta_{b})\;\text{exp}\;\left(\eta_{b}\sum_{j∼i}1(c_{i}=c_{j})\right),$$

where $C(\eta_{b})$ is a normalization constant as long as the batch-specific smoothness parameter $\eta_{b}$ is fixed beforehand, the notation j∼i denotes all spots connected to spot *i* in the graph G, representing the neighbors of spot *i*, and $1(c_{i}=c_{j})$ is the indicator function that equals one whenever $c_{i}=c_{j}$ and zero otherwise. The Hamiltonian or energy function of the Potts prior (5),

$$E(c)=-\sum_{b\;=1}^{B}\sum_{i\;=1}^{n_{b}}\sum_{j∼i}\eta_{b}1(c_{i}{\;=c}_{j}),$$

seeks to minimize the discontinuities among neighboring locations by penalizing differences in label assignments c for adjacent spots in the graph G. This encourages adjacent spots to have the same cluster or spatial domain.

### 2.4 Prior formulation

To account for uncertainty in parameter estimates, priors are set for all parameters in this subsection before Bayesian inference (see Supplementary Material for details). For the parameters in Equation (4), weak priors are used to allow for the fact that the data are pretty much nailed down to posterior distributions,

$$\mu_{k}∼N_{d}(\mu_{\mu },\Sigma_{\mu }),\Lambda ∼W_{d}(n_{\Lambda },\Sigma_{\Lambda }),\omega_{bi}∼G\left(\nu_{\omega }/2,\nu_{\omega }/2\right),$$

where $W_{d}(\cdot )$ is the *d*-dimensional Wishart distribution. By default, we set $\mu_{\mu }=0$ as a consequence of data centering, and the covariance matrix $\Sigma_{\mu }=100\times I_{d}$ to down-weight prior to the posterior mean of $\mu_{k}$. The degree of freedom $n_{\Lambda }$ of the Wishart distribution that satisfies $n_{\Lambda }>d-1$ determines the certainty of the prior information in the scale matrix, and we set $n_{\Lambda }=d$ to provide the least informative specification (Schuurman *et al.* 2016). The scale matrix is set to $\Sigma_{\Lambda }=100\times I_{d}$. $\nu_{\omega }=2$ seems to be successful in avoiding the influence of noise and outliers during segmentation, as set by Gottardo *et al.* (2006).

Focusing now on Equation (3), the priors for $\gamma_{b}$ and $t_{bj}$, the *j*th diagonal of the precision matrix $T_{b}$, are given respectively by

$$\gamma_{b}∼N_{p}(0,I_{p}),t_{bj}∼G\left(\nu_{t}/2,\nu_{t}/2\right),$$

where $\nu_{t}=1$. In other words, the additive batch effects $\gamma_{b}$ are considered white noise in our work. For factor-specific gene selection, a traditional Bayesian approach, called spike-and-slab prior, together with the nonlocal product moment (pMOM) prior (Johnson and Rossell 2010, 2012), which satisfies the additional constraint of vanishing probability at point 0, is adopted for L,

$$L_{ij}|s_{ij}∼(1-s_{ij})N(0,\lambda_{0})+s_{ij}L_{ij}^{2}/\lambda_{1}N(0,\lambda_{1}),$$

where $s_{ij}\in \{0,1\}$, the variances $\lambda_{0}=0.015$ and $\lambda_{1}=0.871$ are fixed beforehand in all our experiments because $P(|L_{ij}|⩽\sqrt{0.1}|\lambda_{0})=0.99$ and $P(|L_{ij}|⩾\sqrt{0.1}|\lambda_{1})=0.99$. Furthermore, a hierarchical prior is set over the indicator $s_{ij}$,

$$s_{ij}|p_{j}∼\text{Bern}(p_{j}),\text{with}\;p_{j}∼B(\alpha_{p},\beta_{p}),$$

where Bern(·) and B(·) denote the Bernoulli and Beta distributions, respectively, and with default hyperparameters $\alpha_{p}=\beta_{p}=1$. All parameters and their hyperparameters are listed in Table 1, and other hyperparameters can be found in Table 2.

**Table 1.** Parameters with their descriptions and hyperparameters.

Parameter	Description	Hyperparameter
$L\in R^{p\times d}$	Loading matrix	$\left(\lambda_{0},\lambda_{1}\right)$
$\gamma \in R^{p\times B}$	Additive batch-effect vector	
$T\in R^{p\times B}$	Multiplicative batch effect, precision matrix of error term	$\nu_{\tau }$
$\mu \in R^{d\times q}$	Mean matrix of latent representations	$\left(\mu_{\mu },\Sigma_{\mu }\right)$
$\omega \in R^{n}$	Sample-specific precision of latent representations	$\nu_{\omega }$
$\Lambda \in R^{d\times d}$	Shared precision matrix of latent representations	$\left(n_{\Lambda },\Sigma_{\Lambda }\right)$
$p\in R^{p}$	Probability in the pMOM prior	$\left(\alpha_{p},\beta_{p}\right)$

**Table 2.** Other hyperparameters in the model.

Description	Hyperparameter
Smoothing parameter of the Potts model	η
Dimension of the lower representation	*d*
Number of clusters	*q*

## 3 Results

### 3.1 STADIA enables more accurate correction of batch effects in the human dorsolateral prefrontal cortex dataset

**Figure 2.**
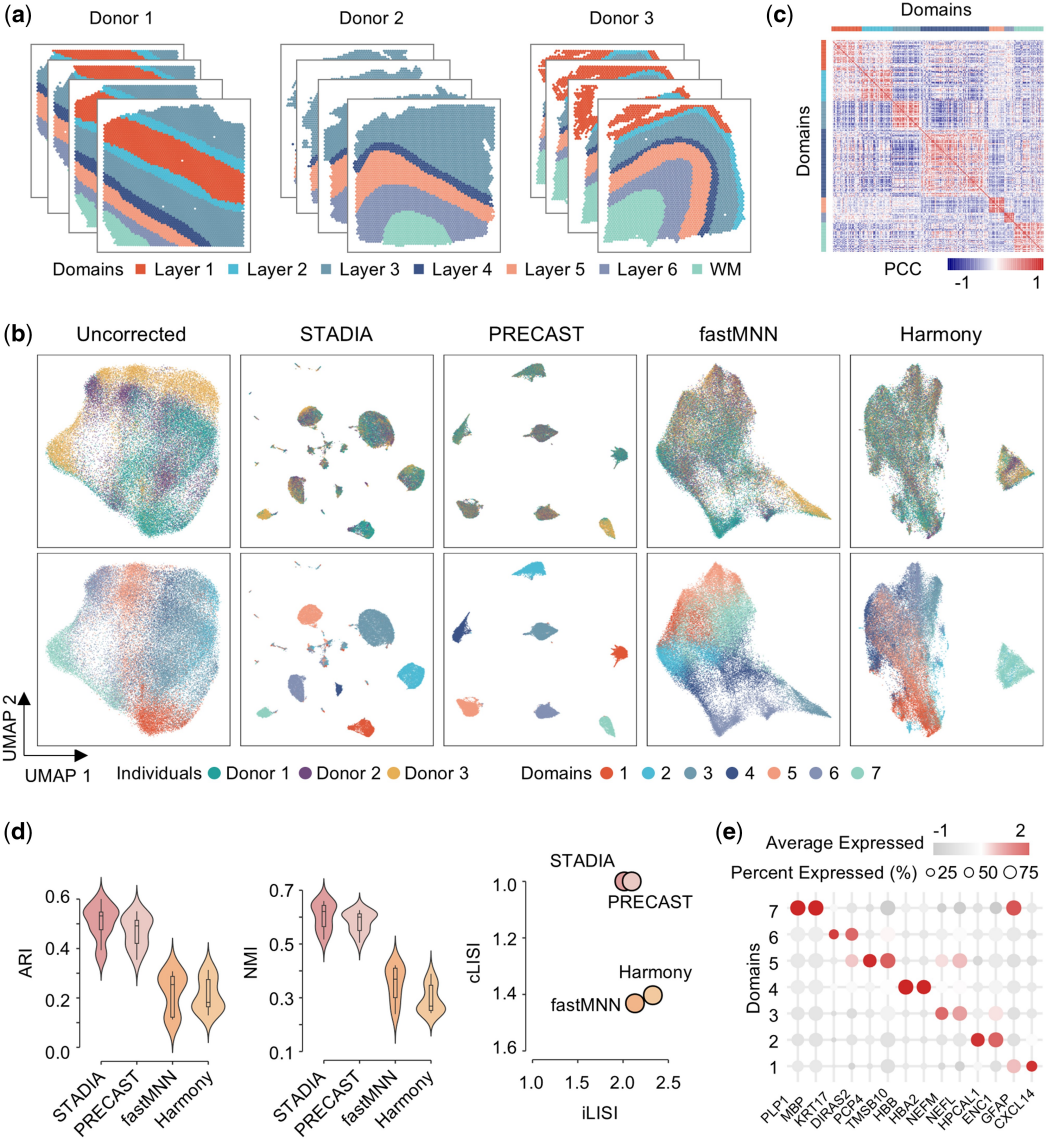
STADIA allows more accurate identification of layer structures and correction of batch effects in the human dorsolateral prefrontal cortex data set. (a) Manual annotation of 12 slices from three donors based on cytoarchitecture labeled by layers 1 to 6 and white matter (WM). (b) Uniform manifold approximation and projection (UMAP) visualization of the original data without correction, STADIA, PRECAST, fastMNN, and Harmony, colored by the donor (top panel) and cluster assignments identified by the corresponding methods (bottom panel). (c) Heatmap of Pearson’s correlation of the gene expressions between different spatial domains identified by STADIA. (d) Violin plots of clustering accuracy in terms of ARI and UMI for the four methods (left and middle panels); scatter plot of mixing scores in terms of LISI, with batch mixing score along the *x*-axis and spatial domain mixing score along the *y*-axis (right panel). A point closer to the upper right corner indicates better performance. (e) Dot plot of the top two marker genes for each spatial domain found by the Wilcoxon rank-sum test.

To quantitatively evaluate the batch mixing and spatial clustering of STADIA, we first applied it to the human dorsolateral prefrontal cortex (DLPFC) dataset measured by the 10x Genomics Visium (Maynard *et al.* 2021). There are a total of 12 tissue slices from three independent neurotypical adult donors (Fig. 2a), each with two pairs of spatially adjacent replicates per adult (four slices per donor). All slices were manually labeled as layers 1 to 6 and white matter (WM) based on cytoarchitecture and genetic markers in the original publication, which will be used as ground truth to evaluate clustering accuracy.

From the Uniform Manifold Approximation and Projection (UMAP) plot of the uncorrected raw data, there are substantial batch effects for the three different donors and negligible batch effects for the slices from the same donor (Fig. 2b, upper left panel and Supplementary Fig. S1a). Therefore, we integrated all 12 slices using STADIA, PRECAST, and two commonly used batch effect correction strategies developed for scRNA-seq data, fastMNN, and Harmony. From the embedded UMAP plots of these four methods, they all mixed the 12 slices well and had comparable Local Inverse Simpson’s Index (LISI) values (Fig. 2b, top panel and Fig. 2d, *x***-**axis of the right panel). Moreover, STADIA and PRECAST yielded more discriminative clusters than fastMNN and Harmony (Fig. 2b, bottom panel and Fig. 2d, *y*-axis of the right panel), indicating that STADIA and PRECAST are adept at preserving the internal structure of the data while effectively distinguishing between different clusters. Next, comparing the spatial domains identified by the four methods with the ground truth, STADIA had the highest clustering accuracy in terms of Adjusted Rand Index (ARI) and Normalized Mutual Information (NMI), with a median ARI of 0.531 and a median NMI of 0.630, which were higher than PRECAST (median ARI = 0.492 and median NMI = 0.600), fastMNN (median ARI = 0.260 and median NMI = 0.367) and Harmony (median ARI = 0.161 and median NMI = 0.263) (Fig. 2d, left and middle panel). Furthermore, spatial visualization of all slices demonstrated that STADIA exhibited more consistent spatial domains compared to alternative methods. Additionally, STADIA showcased smoother layer boundaries (Supplementary Fig. S1b), aligning more consistently with the coherence and structural integrity of the cortical hierarchy. We also calculated Pearson correlations of all clusters to verify the plausibility of the spatial domains identified by STADIA (Fig. 2c). Finally, by comparing each layer with all other layers using the Wilcoxon rank-sum test, we identified marker genes that were differentially expressed in each layer, including previously published markers: *HPCAL1* and *ENC1* for layer 2, *PCP4* for layer 5, *KRT17* for layer 6, and *MBP* for WM (Maynard *et al.* 2021) (Fig. 2e and Supplementary Fig. S1c).

To evaluate the performance of STADIA in SVG selection, we performed differential expression analysis on the results of STADIA to detect domain-specific SVGs and compared the results with those detected by SpatialDE (Svensson *et al.* 2018) and SPARK-X (Zhu *et al.* 2021) (Supplementary Fig. S6a–d). Furthermore, to assess the robustness of the gene sets used as inputs in STADIA, we used the top 2000 HVGs and top 2000 SVGs obtained by SpatialDE and SPARK-X separately and then compared the clustering accuracy in terms of ARI (Supplementary Fig. S6e).

### 3.2 STADIA enables the horizontal integration of two adjacent sagittal mouse brain slices while preserving slice-specific biological variation

**Figure 3.**
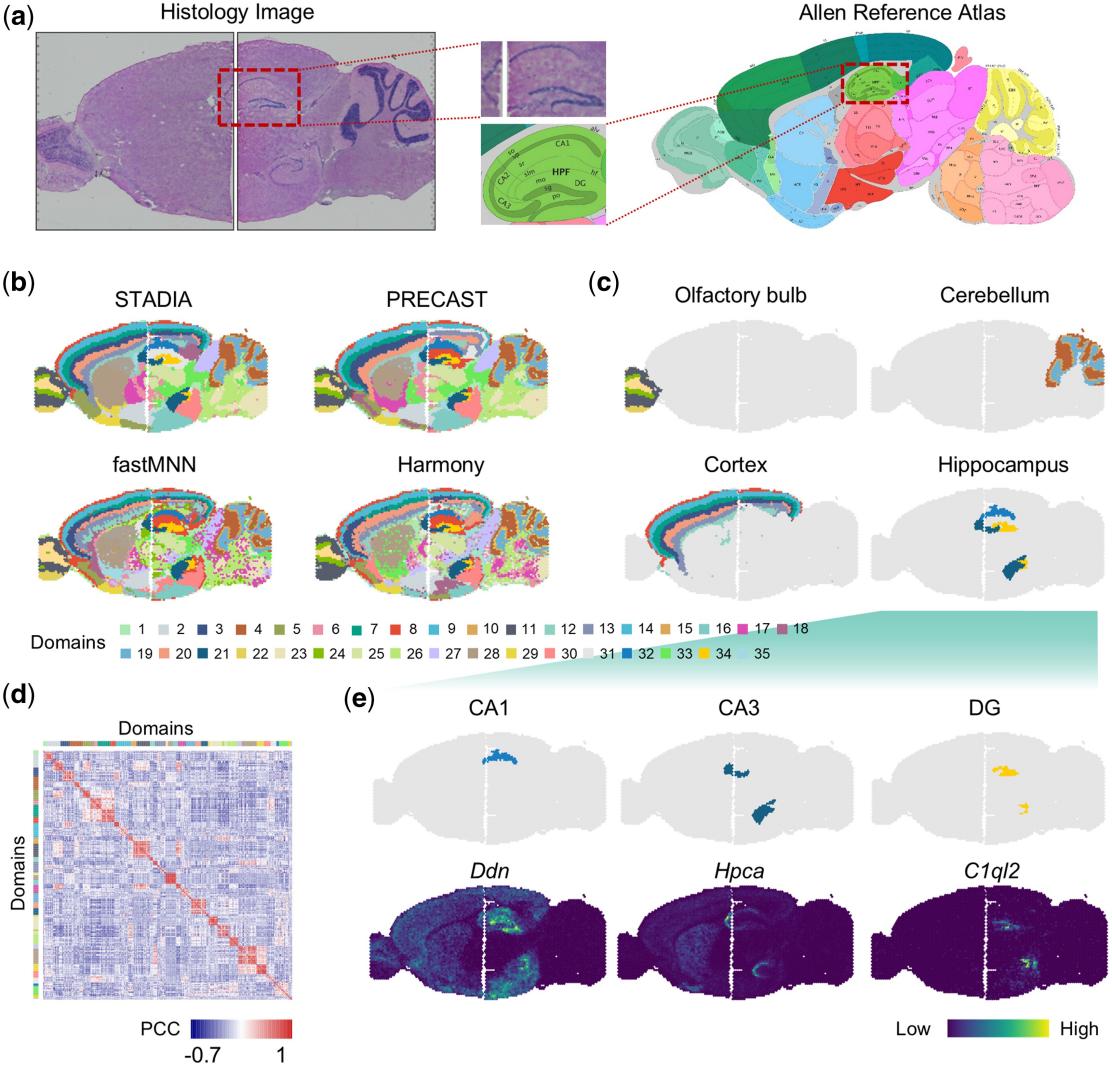
STADIA enables the horizontal integration of two adjacent mouse brain slices while preserving slice-specific biological variation. (a) The Hematoxylin and eosin (H&E) images of two slices and the corresponding tissue structures obtained from the Allen Mouse Brain Atlas. (b) Horizontal alignment of spatial domains of adjacent mouse brain slices, identified by STADIA, PRECAST, fastMNN, and Harmony, respectively. (c) Spatial visualization of slice-specific tissue structures olfactory bulb and cerebellum (top panel), slice-sharing tissue structures cortex and hippocampus (bottom panel), learned by STADIA. (d) Heatmap of Pearson’s correlation of spatial domains identified by STADIA. (e) Visualization of hippocampal subregions learned by STADIA and their corresponding top one marker genes found by the Wilcoxon rank-sum test.

In addition to multiple duplicate slices of a single section, there are many repetitions of horizontally adjacent slices in an experiment, such as data from sagittal mouse brain slices sequenced with the 10x Visium platform. This dataset consists of an anterior sagittal slice and a posterior sagittal slice. We first displayed the hematoxylin & eosin (H&E) stained images corresponding to sagittal mouse brain slices and the tissue structure of the Allen Mouse Brain Atlas (Fig. 3a) and zoomed in on the known hippocampus.

From the full view, STADIA identified commonly known layer structures and horizontally aligned cluster assignments well across two slices (Fig. 3b, upper left panel), such as the shared organizational structures of the cerebral cortex layer (domains 3, 7, 8, 9, 13, and 20) and the hippocampus (domains 21, 32, and 34) (Fig. 3c, bottom panel). This was further validated by the expression of cluster-specific markers *Ddn*, *Hpca*, and *C1ql2* of the hippocampal subregions cornu ammonis 1 (CA1), cornu ammonis 3 (CA3), and dentate gyrus (DG), respectively (Fig. 3e). While identifying the shared tissue structures, STADIA also preserved the slice-specific biological variations, such as the slice-specific tissue structures *olfactory bulb* and *cerebellum* (Fig. 3c, top panel). In comparison, the spatial partitioning of fastMNN and Harmony showed considerable noise, no clear layer boundaries, and PRECAST did not well align domains between the two slices, such as the cortex (Fig. 3b). To further evaluate STADIA, we calculated Pearson correlations between all domains (Fig. 3d), demonstrating a significantly higher within-group similarity compared to between-group similarity.

Furthermore, we compared the alignment of STADIA with that of STAGATE and GraphST demonstrating the critical need and advantage of joint modeling (see Supplementary Fig. S7).

### 3.3 STADIA enables the detection of person-specific cancer domains verified by cancer-associated markers

**Figure 4.**
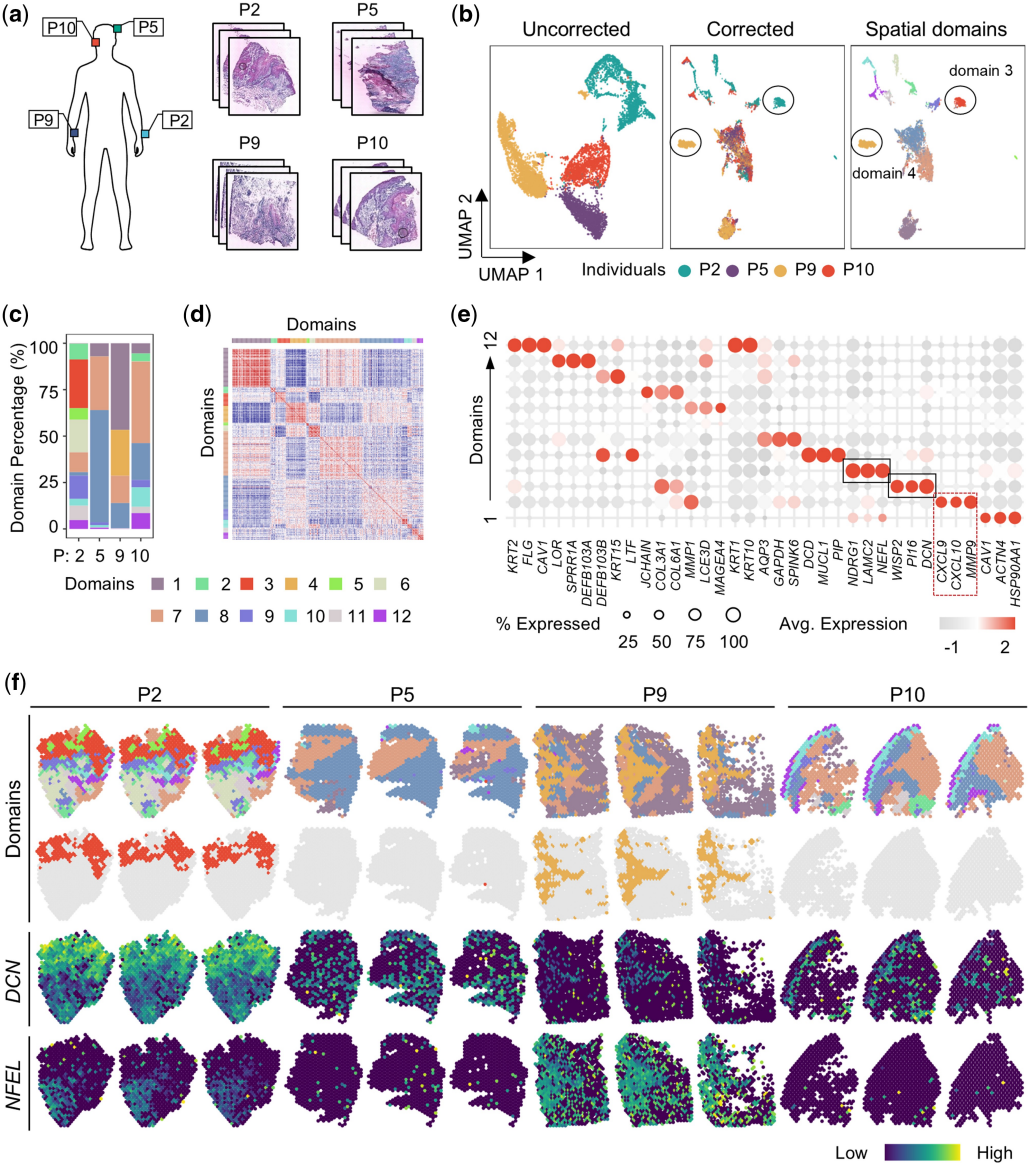
STADIA enables the detection of person-specific cancer domains verified by cancer-associated markers in the cSCC dataset. (a) Hematoxylin and eosin (H&E) images of 12 cSCC slices from different parts of four patients. The top-left three slices were from the left forearm of P2, the top-right three slices were from the left vertex scalp of P5, the bottom-left three slices were from the right forearm of P9, and the bottom-right three slices were from the right tragus of P10. (b) UMAP plots of the original data without correction colored by patients (left panel), and embeddings for STADIA colored by patients (middle panel) and cluster assignments (right panel). (c) Percentage distribution of spatial domains for the four patients, colored as in the left panel of (b). (d) Heatmap of Pearson’s correlation of the gene expressions between different spatial domains identified by STADIA. (e) Dot plot of the top three markers for each spatial domain identified by STADIA. (f) Visualization of spatial domains identified by STADIA in a spatial context (top panel), with domains 3 and 4 highlighted (second panel) and spatial visualization of the corresponding marker genes *DCN* for domain 3 (overexpressed in patient P2) and *NEEL* for domain 4 (overexpressed in P9) (right panel).

Despite having the same type of cancer, patients show very different symptoms. In this section, we studied the human cutaneous squamous cell carcinoma (cSCC) dataset (Ji *et al.* 2020) processed according to the ST protocol (Ståhl *et al.* 2016). It consisted of 12 slices from different parts of four patients, with three cryosections per patient. Specifically, these samples were taken from the left forearm, left vertex scalp, right forearm, and right tragus of each individual (Fig. 4a).

From the UMAP plot of the uncorrected raw data, there was little overlap in these four patients, but the batch effect between the different slices of each patient is not significant (Fig. 4b, left panel and Supplementary Fig. S2a). Based on prior knowledge of the cancers, the lack of overlap may be due to tumor heterogeneity and some degree of batch effects. After correction by STADIA, the embeddings were well mixed, while there were some isolated domains for the P2 and P9 patients, such as domain 3 for P2 and domain 4 for P9 (Fig. 4b, middle and right panels).

Although all slices were cSCC samples, the domain composition of these four patients varied considerably (Fig. 4c), suggesting that these patients may be at different stages of disease progression or have different cancer subtypes. To further explore the correlation of these domains, we calculated the Pearson correlations across all domains (Fig. 4d). The samples from patient P9 mainly consisted of domain 1 and domain 4, which were negatively correlated (Fig. 4d). The most significantly differentially expressed gene in domain 1 was *HSP90AA1* (Fig. 4e), which is associated with disease progression and potential clinical targets for SCC patients (Fan *et al.* 2020). In particular, domain 4 was uniquely present in P9, which was confirmed by the marker gene *NEFL* (Fig. 4e and f). Similarly, domain 3 appeared only in P2 with high expression of the gene *DCN* (Fig. 4e and f) and expressed markers of fibroblasts such as *PI16* and *WISP2*.

Previous studies have shown that the expression of both *DCN* and *NEFL* correlates with the invasive ability of cancer cells and that high levels of *DCN* and *NEFL* decrease the invasive ability of cancer cells (Huang *et al.* 2014, Hu *et al.* 2021b). In contrast, highly expressed marker genes for domain 2 were therapeutic targets for related human malignancies, such as *MMP9* (Augoff *et al.* 2022, Tufaro *et al.* 2011), *CXCL10* (Liu *et al.* 2011), and *CXCL9* (Ding *et al.* 2016), suggesting that domain 2 is a relatively severe tumor region (Fig. 4e). Furthermore, from the stacked bar plot of cell-type composition, we can directly conclude that domain 2 was predominantly found in P2 and P10 patients (Fig. 4c). To refine our analysis, we visualized the expression patterns of the top three markers within domain 2 in a spatial context. The results showed that *MMP9* was highly expressed in domain 2 of both P2 and P10, while *CXCL10* and *CXCL9* exhibited elevated expression levels exclusively in domain 2 of P2 (Supplementary Fig. S2b). This observation was further confirmed by the violin plots of the expression of these three genes (Supplementary Fig. S2c). Such distinctions in expression profiles may suggest the presence of distinct cellular subtypes or functional subtypes within domain 2 in these two patients, which could be associated with different characteristics or clinical factors of the tumor.

We further applied STADIA to the mouse liver dataset (Hildebrandt *et al.* 2021), which consists of eight liver tissue slices from three adult female wild mice profiled by the ST protocol. Our analysis successfully identified the central vein (CV) and portal vein (PV) across all eight slices, revealing a negative correlation in marker gene expression between these anatomical structures (in Supplementary Material: “Further Analysis” section and Fig. S5).

### 3.4 STADIA allows identification of common hippocampal tissue structures while preserving slice-specific biological variation by integrating two hippocampal slices from slide-seqV2

To demonstrate the scalability of STADIA to different spatial resolutions, we applied STADIA to a mouse hippocampus dataset profiled by slide-seqV2 (Stickels *et al.* 2021), which can profile spatial expression at near-cellular resolution (10 µm). This dataset consists of two slices with batch effects present (Supplementary Fig. S4a, upper left panel).

**Figure 5.**
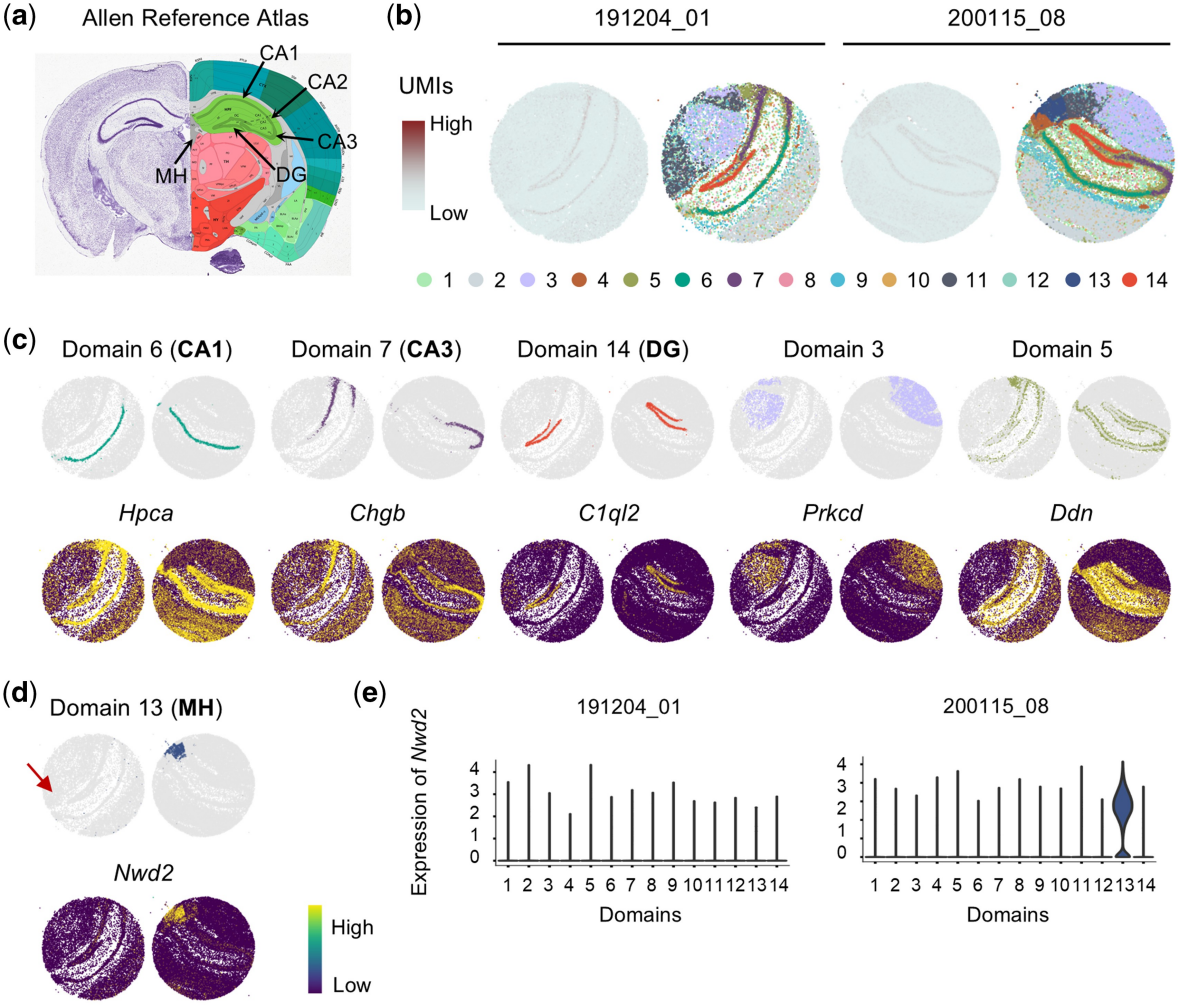
STADIA enables the identification of common hippocampal tissue structures while preserving slice-specific variation by integrating two hippocampal slices from Slide-seqV2. (a) The reference tissue structures from the Allen Mouse Brain Atlas. (b) Spatial visualization for two slices, colored by the number of UMIs and spatial domains identified by STADIA. (c) Spatial visualization of domains 6, 7, 14, 3, and 5 identified by STADIA with their top marker genes found by the Wilcoxon rank-sum test. (d) The slice-specific domain 13 identified by STADIA for the second slice, marked by the gene *Nwd2*. (e) The violin plots for the expression of the gene *Nwd2* of two slices. (f) Histogram of the log2-transformed number of spots and UMIs for two slices, with vertical lines corresponding to the gene *Nwd2*.

From the Allen Reference Atlas, we can see that the hippocampal tissue consists of the cornu ammonis (CA) with subregions CA1, CA2, and CA3, and the dentate gyrus (DG) (Fig. 5a). As expected, STADIA removed batch effects of the two slices (Supplementary Fig. S4a) and successfully characterized the hippocampal structures for both slices with clear domain boundaries (Fig. 5b). In addition, the domain-specific marker genes were found by the Wilcoxon rank-sum test, and five of all common domains together with their most significant marker genes were plotted in a spatial context (Fig. 5c). For example, the expression of *C1ql2* showed a clear arrowhead shape that was highly expressed in the hippocampal subregion DG and the expression of *Ddn* was highly expressed around the structure of the hippocampus corresponding to domain 5.

Furthermore, a slice-specific domain (domain 13) was identified by STADIA for the second slice (puck_200115_08), which was characterized by the marker gene *Nwd2* (Fig. 5d). We found that *Nwd2* was highly expressed only in domain 13 of the second slice (Fig. 5e). This underscores STADIA’s ability to maintain the distinct biological variations of individual slices while uncovering shared biological properties across multiple slices.

Additionally, to further evaluate STADIA’s ability to handle different scales of batch effects, we conducted simulation studies that included two scenarios, each with small, medium, and large batch effects. These scenarios used gene expression profiles simulated from Gaussian mixture models (GMM) with spatial locations and domains derived from DLPFC datasets. Please refer to the “Evaluation with simulated data” subsection in Supplementary Material and Supplementary Fig. S8.

## 4 Discussion

Due to the limitations of existing technologies, a single experiment can only capture limited biological signals in a small region. With the rapid accumulation of ST data, we developed a hierarchical hidden Markov random field model STADIA to align spots from multiple ST slices with batch-effect correction. The observed expression profiles and the batch-corrected embeddings are linked by a Bayesian factor regression model with L/S adjustment. The batch-corrected embeddings and the cluster assignments are linked by a GMM model, with the cluster assignments further modeled by the Potts model to ensure local smoothing. Through extensive experiments, STADIA can successfully mix multiple ST slices without overcorrection and preserve slice-specific biological variations. Compared to PRECAST, STADIA achieves competitive results using a more straightforward model. In addition, STADIA further improves the alignment between slices by connecting MNN pairs across multiple slices when constructing the neighborhood graph.

The main limitation is that the linear dimensionality reduction we used results in more data loss than a nonlinear method. Future work is expected to employ nonlinear methods such as an autoencoder, which links the observed expression to hidden embeddings, and Markov random field model-driven clustering methods for further spatially aware embedded clustering.

STADIA is implemented in R. Although we have used OpenMP for parallel acceleration in large matrix operations, the time complexity remains linear in the number of locations. We also need to optimize memory usage to improve performance, including the input data type of the algorithm (deleting the parts of the Seurat object that are not used in the algorithm) (see Supplementary Table S5 for details on the running time and memory usage for the five experiments conducted above.).

Our current work is limited to transcriptome analysis. In the future, we plan to jointly analyze muti-omics data across multiple slices, which can provide insights into the regulation of the entire process, spanning from gene to protein expression. To address this issue, we need to consider the batch effects across different slices as well as the regulatory mechanisms of various omics, which poses a greater challenge.

## Supplementary Material

btae611_Supplementary_Data

## Data Availability

All datasets analyzed in this study are available through websites reported in the original publications. Please refer to the Supplementary Material for the specific download links. The STADIA algorithm is implemented using R software and is packaged as an R package *stadia*, which is available at https://github.com/zhanglabtools/STADIA and https://zenodo.org/records/13637744.
