# Supplementary material for: Statistical batch-aware embedded integration, dimension reduction, and alignment for spatial transcriptomics
Source: Bioinformatics. 2024 Oct 14;40(10):btae611. doi: 10.1093/bioinformatics/btae611 (PMC11512591; doi:10.1093/bioinformatics/btae611)
Supplement: btae611_Supplementary_Data [file btae611_supplementary_data.zip › supplementary.pdf]

Supplementary Materials for

Statistical batch-aware embedded integration, dimension reduction and alignment for spatial transcriptomics

Yanfang Li[1] and Shihua Zhang[1,2,3*]

[1]NCMIS, CEMS, RCSDS, Academy of Mathematics and Systems Science, Chinese Academy of Sciences, Beijing 100190, China
[2]School of Mathematical Sciences, University of Chinese Academy of Sciences, Beijing 100049, China
[3]Key Laboratory of Systems Health Science of Zhejiang Province, School of Life Science, Hangzhou Institute for Advanced Study, University of Chinese Academy of Sciences, Hangzhou, 310024, China

* To whom correspondence should be addressed. Tel/Fax: +86 01 82541360; Email zsh@amss.ac.cn

Supplementary Notes

Marginal student-t distribution [Liu and Rubin, 1995]

Multivariate t-distribution $t_d(\nu, \boldsymbol{\mu}, \boldsymbol{\Sigma})$ is a multivariate probability distribution with density

$$f_t(\boldsymbol{x}; \nu, \boldsymbol{\mu}, \boldsymbol{\Sigma}) = \frac{\Gamma\left(\frac{\nu+d}{2}\right)}{\Gamma\left(\frac{\nu}{2}\right)(\nu\pi)^{\frac{d}{2}} |\boldsymbol{\Sigma}|^{\frac{1}{2}}} \left[1 + \frac{1}{\nu}(\boldsymbol{x} - \boldsymbol{\mu})^\top \boldsymbol{\Sigma}^{-1}(\boldsymbol{x} - \boldsymbol{\mu})\right]^{-\frac{\nu+d}{2}}.$$

Lemma 0.1. *When given the weight τ, $\boldsymbol{x}$ has the multivariate normal distribution*

$$\boldsymbol{x} \mid \tau \sim \mathcal{N}_p(\boldsymbol{\mu}, \tau^{-1}\boldsymbol{\Sigma}),$$

and $\tau\nu$ is χ^2_ν, that is, the weight τ is Gamma distribution given ν

$$\tau \mid \nu \sim \textit{Gamma}(\nu/2, \nu/2),$$

then the marginal distribution of $\boldsymbol{x}$ by integrating out τ is multivariate student-t distribution $t_p(\nu, \boldsymbol{\mu}, \boldsymbol{\Sigma})$.

Motivation of L/S adjustment

The Location-and-Scale (L/S) adjustment is the cornerstone of the ComBat algorithm applied to microarray data for batch effect correction, which works by estimating batch-specific location and scale parameters for each gene or feature across various batches. Similarly, STADIA adopts a similar strategy to harmonize gene expression across different batches within spatial transcriptomics (ST) datasets. Here, we outline the rationale behind the L/S adjustment from two aspects.

1. Experimental aspect: Systematic differences between batches, such as variations in instrument calibration and batches of reagents, can lead to an overall shift in the measurement results. Additionally, factors like experimental platforms and instrument sensitivity may cause changes in data proportions, even when measuring the same feature in the same samples. The L/S adjustment addresses these challenges by employing additive and multiplicative corrections. It adds a batch-specific bias and scaling factor to each variable, effectively correcting for shifts and proportional changes induced by the batch effects.

2. Data distribution aspect: After normalization, data generally follows a normal distribution, uniquely determined by its mean and covariance matrix. The mean represents the central tendency of the distribution, while the covariance matrix measures the variation of the randomness. To harmonize the gene expressions across different batches, which are assumed to be normally distributed, adjusting the means and covariance matrices is sufficient.

Determining the number of clusters

All numbers of clusters in the experiments are set to be the same as those in the original publication, except for the mouse brain samples. For the mouse brain samples, the number of clusters used was the smallest required to align the embeddings from STADIA. For other data, if biological prior knowledge is available, we recommend using it to determine the number of clusters. Traditional statistical methods, such as AIC and BIC, can be employed to identify the number of clusters without such prior knowledge.

Principal to build the spatial neighborhood graph

For ST and Visium data arranged on square and hexagonal lattices, we defined neighborhood sets based on shared edges. For other data, we defined neighborhood sets using a predefined radius r to ensure that each spot has an average of 6-15 neighbors.

Overall framework of Bayesian inference

First, all parameters and all latent or missing variables are combined into $\theta = \{\mathbf{L}, \mathbf{T}, \mathbf{\Lambda}, \boldsymbol{\gamma}, \boldsymbol{\mu}, \boldsymbol{\omega}, \mathbf{p}\}$ and $\mathbf{Z} = \{\boldsymbol{f}, \boldsymbol{c}, \boldsymbol{s}\}$ respectively. The Expectation-Maximization (EM) algorithm is then used to obtain maximum a posteriori (MAP) estimates for all parameters. By standard algebraic calculations with Jensen's Inequality, a tight lower bound for the log posterior is given by

$$\log\left[\mathrm{P}(\theta \mid \mathbf{X})\right] \geqslant \mathbb{E}_{z|x,\theta^{(t)}} \log\left[\mathrm{P}(\theta \mid \mathbf{X}, \mathbf{Z})\right] + C \overset{\text{def}}{=} Q(\theta \mid \theta^{(t)}) + C, \tag{0.1}$$

where $\mathbb{E}_{z|x,\theta^{(t)}}$ denotes the expectation of the latent variable $\mathbf{Z}$ conditional on the observation $\mathbf{X}$ and the current values of parameters $\theta^{(t)}$, C is a constant with respect to θ, and all of the following C's are constants whose values vary from place to place. The EM algorithm then iteratively solves the following optimization problem

$$\theta^{(t+1)} = \arg\max_{\theta} Q(\theta \mid \theta^{(t)}).$$

In the expectation step, the core objective is to obtain an explicit notation for $Q(\theta \mid \theta^{(t)})$, by computing the two conditional distributions $\mathrm{P}(\theta \mid \mathbf{X}, \mathbf{Z})$ and $\mathrm{P}(\mathbf{Z} \mid \mathbf{X}, \theta^{(t)})$. From the conditional independence of $\{\boldsymbol{f}, \boldsymbol{c}, \boldsymbol{s}\}$ given others, we have

$$\begin{aligned}\mathrm{P}(\mathbf{Z} \mid \mathbf{X}, \theta^{(t)}) &= \prod_{b,i} \mathrm{P}(\boldsymbol{f}_{bi} \mid c_{bi}, \boldsymbol{x}_{bi}, \theta^{(t)}) \mathrm{P}(\boldsymbol{c} \mid \mathbf{X}, \theta^{(t)}) \mathrm{P}(\boldsymbol{s} \mid \theta^{(t)}) \\ &\approx \underbrace{\prod_{b,i} \mathrm{P}(\boldsymbol{f}_{bi} \mid c_{bi}, \boldsymbol{x}_{bi}, \theta^{(t)})}_{P_{11}} \underbrace{\prod_{b,i} \mathrm{P}(c_{bi} \mid \hat{\boldsymbol{c}}_{\mathsf{N}_{bi}}, \boldsymbol{x}_{bi}, \theta^{(t)})}_{\widetilde{P}_{12}} \underbrace{\mathrm{P}(\boldsymbol{s} \mid \theta^{(t)})}_{P_2},\end{aligned} \tag{0.2}$$

where the last approximation sign $\approx$ is due to the spatial dependence of the $\boldsymbol{c}$ on each other,

and a pseudo probability is used. Then, from Bayes' formula, the following equation holds

$$\begin{aligned}
&\log\left[\mathrm{P}(\theta \,|\, \mathbf{X}, \mathbf{Z})\right] \\
&= \log\left[\mathrm{P}(\mathbf{X}, \boldsymbol{f}, \boldsymbol{c} \,|\, \mathbf{L}, \mathbf{T}, \boldsymbol{\Lambda}, \boldsymbol{\gamma}, \boldsymbol{\mu}, \boldsymbol{\omega})\right] + \log\left[\mathrm{P}(\mathbf{L}, \boldsymbol{s} \,|\, \boldsymbol{p})\right] + \log\left[\pi(\mathbf{T})\pi(\boldsymbol{\Lambda})\pi(\boldsymbol{\gamma})\pi(\boldsymbol{\mu})\pi(\boldsymbol{\omega})\pi(\boldsymbol{p})\right] + C \\
&\approx \underbrace{\log \prod_{b,i} \mathrm{P}(\boldsymbol{x}_{bi}, \boldsymbol{f}_{bi}, c_{bi} \,|\, \hat{\boldsymbol{c}}_{\mathrm{N}_{bi}}, \mathbf{L}, \mathbf{T}_b, \boldsymbol{\Lambda}, \boldsymbol{\gamma}_b, \boldsymbol{\mu}, \omega_{bi})}_{\widetilde{Q}_1} + \underbrace{\log\left[\mathrm{P}(\mathbf{L}, \boldsymbol{s} \,|\, \boldsymbol{p})\right]}_{Q_2} + \underbrace{\log\left[\pi(\mathbf{T})\pi(\boldsymbol{\Lambda})\pi(\boldsymbol{\gamma})\pi(\boldsymbol{\mu})\pi(\boldsymbol{\omega})\pi(\boldsymbol{p})\right]}_{Q_3} + C,
\end{aligned} \tag{0.3}$$

where $\widetilde{Q}_1$ is a pseudo approximation of the complete-data log-likelihood corresponding to $(\mathbf{X}, \boldsymbol{f}, \boldsymbol{c})$ for the same reason as (0.2), Q_2 is the complete-data log-likelihood corresponding to the parameter $\mathbf{L}$ and Q_3 are joint priors. Finally, substituting (0.2) and (0.3) into $Q(\theta \,|\, \theta^{(t)})$, we get

$$\widetilde{Q}(\theta \,|\, \theta^{(t)}) = \mathbb{E}_{\widetilde{P}_1}[\widetilde{Q}_1] + \mathbb{E}_{P_2}[Q_2] + Q_3 + C, \tag{0.4}$$

which is a pseudo probability of $Q(\theta \,|\, \theta^{(t)})$ and where $\widetilde{P}_1 = P_{11}\widetilde{P}_{12}$.

In the maximization step, the block gradient descent algorithm is used to optimize $\widetilde{Q}(\theta \,|\, \theta^{(t)})$.

$$\begin{aligned}
\mathbf{T}_b^{(t+1)} &= \arg\max_{T_b} \widetilde{Q}(T_b \,|\, \theta^{(t)}_{\backslash T_b}), \quad \text{for } b = 1, 2, \cdots, B, \\
\boldsymbol{\gamma}_b^{(t+1)} &= \arg\max_{\gamma_b} \widetilde{Q}(\gamma_b \,|\, \theta^{(t)}_{\backslash \gamma_b}), \quad \text{for } b = 1, 2, \cdots, B, \\
\boldsymbol{\mu}_k^{(t+1)} &= \arg\max_{\mu_k} \widetilde{Q}(\boldsymbol{\mu}_k \,|\, \theta^{(t)}_{\backslash \boldsymbol{\mu}_k}), \quad \text{for } k = 1, 2, \cdots, q, \\
\omega_{bi}^{(t+1)} &= \arg\max_{\omega_{bi}} \widetilde{Q}(\omega_{bi} \,|\, \theta^{(t)}_{\backslash \omega_{bi}}), \quad \text{for } b = 1, 2, \cdots, B, \text{ and } i = 1, 2, \cdots, n_b, \\
\boldsymbol{\Lambda}^{(t+1)} &= \arg\max_{\Lambda} \widetilde{Q}(\boldsymbol{\Lambda} \,|\, \theta^{(t)}_{\backslash \boldsymbol{\Lambda}}), \\
p_j^{(t+1)} &= \arg\max_{p_j} \widetilde{Q}(p_j \,|\, \theta^{(t)}_{\backslash p_j}), \quad \text{for } j = 1, 2, \cdots, d, \\
L_{ij}^{(t+1)} &= \arg\max_{L_{ij}} \widetilde{Q}(L_{ij} \,|\, \theta^{(t)}_{\backslash L_{ij}}), \quad \text{for } i = 1, 2, \cdots, p, \text{ and } j = 1, 2, \cdots, d,
\end{aligned} \tag{0.5}$$

where $\theta^{(t)}_{\backslash *}$ means all the parameters except for $*$.

Detailed calculations of Bayesian inference

Calculation of Eq. (0.1) First, based on Bayes' formula, we rewrite the log posterior $\log\,\mathrm{P}(\theta \,|\, \mathbf{X})$,

$$\log\left[\mathrm{P}(\theta \,|\, \mathbf{X})\right] = \log\left[\mathrm{P}(\mathbf{X} \,|\, \theta)\pi(\theta)\right] - \log\left[\mathrm{P}(\mathbf{X})\right]. \tag{0.6}$$

Then, due to the independence of $\mathrm{P}(\mathbf{X})$ with respect to θ, the focus is mainly on the first term,

$$\begin{aligned}
\log\left[\mathrm{P}(\mathbf{X} \,|\theta)\pi(\theta)\right] &= \log\left[\int_z \mathrm{P}(\mathbf{X}, \mathbf{Z} \,|\, \theta)\, \pi(\theta) \mathrm{d}z\right] \\
&= \log\left[\int_z \mathrm{P}\Big(\mathbf{Z} \,|\, \mathbf{X}, \theta^{(t)}\Big) \frac{\mathrm{P}(\mathbf{X}, \mathbf{Z} \,|\, \theta)\, \pi(\theta)}{\mathrm{P}\big(\mathbf{Z} \,|\, \mathbf{X}, \theta^{(t)}\big)} \mathrm{d}z\right]
\end{aligned}$$

$$
\begin{aligned}
&\geqslant \int_z \mathrm{P}\Big(\mathbf{Z}\,|\,\mathbf{X},\theta^{(t)}\Big)\log\left[\frac{\mathrm{P}(\mathbf{X},\mathbf{Z}\,|\,\theta)\,\pi(\theta)}{\mathrm{P}\big(\mathbf{Z}\,|\,\mathbf{X},\theta^{(t)}\big)}\right]\mathrm{d}z \\
&= \mathbb{E}_{z|x,\theta^{(t)}}\log\left[\mathrm{P}(\theta\,|\,\mathbf{X},\mathbf{Z})\right]+C(\theta^{(t)}),
\end{aligned}
\tag{0.7}
$$

where the greater-than sign comes from Jensen's Inequality and $C(\theta^{(t)})$ is a constant with respect to θ. Finally, by substituting (0.7) into (0.6) , we can obtain a lower bound on the log posterior $\log\left[\mathrm{P}(\theta\,|\,\mathbf{X})\right]$

$$
\log\left[\mathrm{P}(\theta\,|\,\mathbf{X})\right]\geqslant \mathbb{E}_{z|x,\theta^{(t)}}\log\left[\mathrm{P}(\theta\,|\,\mathbf{X},\mathbf{Z})\right]+C,
$$

where $C=C(\theta^{(t)})-\log\left[\mathrm{P}(\mathbf{X})\right]$ is also a constant.

Calculation of Eq. (0.2) The latent variable $\mathbf{Z}$ in our model consists of the low-dimensional batch-corrected factor $\boldsymbol{f}$, the cell type indicator $\boldsymbol{c}$, and the spike-and-slab distributional indicator $\boldsymbol{s}$. From the independence of $\boldsymbol{f},\boldsymbol{c}$ and $\boldsymbol{s}$ under other random variables, the conditional distribution Eq. (0.2) could be computed separately, i.e., P_{11}, $\tilde{P}_{12}$ and P_2. Now, we compute P_{11}, $\tilde{P}_{12}$ and P_2 with $\theta^{(t)}$ shorted by θ for briefness,

$$
\begin{aligned}
P_{11} &= \prod_{b,i}\mathrm{P}(\boldsymbol{f}_{bi}\,|\,c_{bi},\boldsymbol{x}_{bi},\theta)=\prod_{b,i,k}\mathrm{P}(\boldsymbol{f}_{bi}\,|\,c_{bi}=k,\boldsymbol{x}_{bi},\theta)^{\mathbb{I}(c_{bi}=k)} \\
&= \prod_{b,i,k}\exp\{\log\left[\mathrm{P}(\boldsymbol{x}_{bi}\,|\,\boldsymbol{f}_{bi},\theta)\right]+\log\left[\mathrm{P}(\boldsymbol{f}_{bi}\,|\,c_{bi}=k,\theta)\right]-\log\left[\mathrm{P}(\boldsymbol{x}_{bi}\,|\,c_{bi}=k,\theta)\right]\}^{\mathbb{I}(c_{bi}=k)} \\
&\propto \prod_{b,i,k}\exp\left\{-\frac{1}{2}\Big[(\boldsymbol{x}_{bi}-\mathbf{L}\boldsymbol{f}_{bi}-\boldsymbol{\gamma}_b)^\top\mathbf{T}_b(\boldsymbol{x}_{bi}-\mathbf{L}\boldsymbol{f}_{bi}-\boldsymbol{\gamma}_b)+(\boldsymbol{f}_{bi}-\boldsymbol{\mu}_k)^\top\omega_{bi}\boldsymbol{\Lambda}(\boldsymbol{f}_{bi}-\boldsymbol{\mu}_k)\Big]\right\}^{\mathbb{I}(c_{bi}=k)} \\
&\propto \prod_{b,i,k}\exp\left\{-\frac{1}{2}\Big[\boldsymbol{f}_{bi}^\top(\mathbf{L}^\top\mathbf{T}_b\mathbf{L}+\omega_{bi}\boldsymbol{\Lambda})\boldsymbol{f}_{bi}-2(\mathbf{L}^\top\mathbf{T}_b\widetilde{\boldsymbol{x}}_{bi}+\omega_{bi}\boldsymbol{\Lambda}\boldsymbol{\mu}_k)^\top\boldsymbol{f}_{bi}\Big]\right\}^{\mathbb{I}(c_{bi}=k)} \\
&= \prod_{b,i,k}\left[\mathcal{N}(\Phi_{bi}^{-1}\varphi_{bik},\Phi_{bi}^{-1})\right]^{\mathbb{I}(c_{bi}=k)}, \\
\widetilde{P}_{12} &= \prod_{b,i}\mathrm{P}(c_{bi}\,|\,\hat{\boldsymbol{c}}_{\mathsf{N}_{bi}},\boldsymbol{x}_{bi},\theta)=\prod_{b,i,k}\left[\mathrm{P}(c_{bi}=k\,|\,\hat{\boldsymbol{c}}_{\mathsf{N}_{bi}},\boldsymbol{x}_{bi},\theta)\right]^{\mathbb{I}(c_{bi}=k)}\propto\prod_{b,i,k}\left[\mathrm{P}(\boldsymbol{x}_{bi},c_{bi}=k\,|\,\hat{\boldsymbol{c}}_{\mathsf{N}_{bi}},\theta)\right]^{\mathbb{I}(c_{bi}=k)} \\
&= \prod_{b,i,k}\left[\int_f\mathrm{P}(\boldsymbol{x}_{bi},\boldsymbol{f}_{bi}\,|\,c_{bi}=k,\theta)\mathrm{d}f\times\mathrm{P}(c_{bi}=k\,|\,\hat{\boldsymbol{c}}_{\mathsf{N}_{bi}})\right]^{\mathbb{I}(c_{bi}=k)} \\
&\propto \prod_{b,i,k}\left\{\exp\left[-\frac{1}{2}(\tilde{\boldsymbol{x}}_{bi}-\Psi_{bi}^{-1}\psi_{bik})^\top\Psi_{bi}(\tilde{\boldsymbol{x}}_{bi}-\Psi_{bi}^{-1}\psi_{bik})+\eta_b\sum_{j\in\mathsf{N}_{bi}}\mathbb{I}(\hat{c}_j=k)\right]\right\}^{\mathbb{I}(c_{bi}=k)} \\
&= \prod_{b,i,k}\left\{\exp\left[-\frac{1}{2}(\tilde{\boldsymbol{x}}_{bi}-\mathbf{L}\boldsymbol{\mu}_k)^\top\Psi_{bi}(\tilde{\boldsymbol{x}}_{bi}-\mathbf{L}\boldsymbol{\mu}_k)+\eta_b\sum_{j\in\mathsf{N}_{bi}}\mathbb{I}(\hat{c}_j=k)\right]\right\}^{\mathbb{I}(c_{bi}=k)} \\
&\propto \prod_{b,i,k}\left[S_{bik}\right]^{\mathbb{I}(c_{bi}=k)}, \\
P_2 &= \mathrm{P}(\boldsymbol{s}\,|\,\theta)=\prod_{i,j}\mathrm{P}(s_{ij}\,|\,L_{ij},p_j)\propto\prod_{i,j}\mathrm{P}(L_{ij}\,|\,s_{ij})\mathrm{P}(s_{ij}\,|\,p_j)
\end{aligned}
$$

$$
\begin{aligned}
=&\Pi_{i,j}\left[\frac{p_j L_{ij}^2}{\lambda_1}|2\pi\lambda_1|^{-1/2}\exp\Big(-\frac{L_{ij}^2}{2\lambda_1}\Big)\right]^{s_{ij}}\left[(1-p_j)|2\pi\lambda_0|^{-1/2}\exp\Big(-\frac{L_{ij}^2}{2\lambda_0}\Big)\right]^{1-s_{ij}}\\
=&\prod_{i,j}[h_{ij}]^{s_{ij}}[1-h_{ij}]^{1-s_{ij}}.
\end{aligned}
$$

where $\propto$ means equal up to a constant, $\tilde{\boldsymbol{x}}_{bi}=\boldsymbol{x}_{bi}-\boldsymbol{\gamma}_b$, $\Phi_{bi}=\mathbf{L}^\top\mathbf{T}_b\mathbf{L}+\omega_{bi}\boldsymbol{\Lambda}$, $\varphi_{bik}=\mathbf{L}^\top\mathbf{T}_b\tilde{\boldsymbol{x}}_{bi}+\omega_{bi}\boldsymbol{\Lambda}\boldsymbol{\mu}_k$, $\Psi_{bi}=\mathbf{T}_b-\mathbf{T}_b\mathbf{L}\Phi_{bi}^{-1}\mathbf{L}^\top\mathbf{T}_b$, $\psi_{bik}=\mathbf{T}_b\mathbf{L}\Phi_{bi}^{-1}\omega_{bi}\boldsymbol{\Lambda}\boldsymbol{\mu}_k$,

$$
h_{ij}=\frac{1}{1+\frac{1-p_j}{p_j}\frac{\lambda_1}{L_{ij}^2}\sqrt{\frac{\lambda_1}{\lambda_0}}\exp\Big(-\frac{L_{ij}^2}{2}\Big(\frac{1}{\lambda_0}-\frac{1}{\lambda_1}\Big)\Big))},
$$

and

$$
S_{bik}\propto\exp\Big[-\frac{1}{2}(\tilde{\boldsymbol{x}}_{bi}-\mathbf{L}\boldsymbol{\mu}_k)^\top\Psi_{bi}(\tilde{\boldsymbol{x}}_{bi}-\mathbf{L}\boldsymbol{\mu}_k)+\eta_b\sum_{j\in\mathsf{N}_{bi}}\mathbb{I}(\hat{c}_j=k)\Big]\quad\text{such that}\quad\sum_k S_{bik}=1.
$$

Calculation of Eq. (0.3) Based on Bayes' formula, the complete-data log posterior $\mathrm{P}(\theta\,|\,\mathbf{X},\mathbf{Z})$ is split into three parts $\widetilde{Q}_1$, Q_2 and Q_3, which will be calculated in turn.

$$
\begin{aligned}
\widetilde{Q}_1=&\log\left[\prod_{b,i}\mathrm{P}(\boldsymbol{x}_{bi},\boldsymbol{f}_{bi},c_{bi}\,|\,\hat{\boldsymbol{c}}_{\mathsf{N}_{bi}},\mathbf{L},\mathbf{T}_b,\boldsymbol{\Lambda},\boldsymbol{\mu},\omega_{bi},\boldsymbol{\gamma}_b)\right]\\
=&\log\left[\prod_{b,i,k}\Big\{\mathrm{P}(\boldsymbol{x}_{bi},\boldsymbol{f}_{bi}\,|\,c_{bi}=k,\mathbf{L},\mathbf{T}_b,\boldsymbol{\Lambda},\boldsymbol{\mu}_{c_{bi}},\omega_{bi},\boldsymbol{\gamma}_{bi})\mathrm{P}(c_{bi}=k\,|\,\hat{\boldsymbol{c}}_{\mathsf{N}_{bi}})\Big\}^{\mathbb{I}(c_{bi}=k)}\right]\\
=&\sum_{b,i,k}\mathbb{I}(c_{bi}=k)\big\{\log\left[\mathrm{P}(\boldsymbol{x}_{bi}\,|\,\boldsymbol{f}_{bi},\mathbf{L},\mathbf{T}_b,\boldsymbol{\gamma}_{bi})\right]+\log\left[\mathrm{P}(\boldsymbol{f}_{bi}\,|\,c_{bi}=k,\boldsymbol{\Lambda},\boldsymbol{\mu}_k,\omega_{bi})\right]+\log\left[\mathrm{P}(c_{bi}=k\,|\,\hat{\boldsymbol{c}}_{\mathsf{N}_{bi}})\right]\big\}\\
=&\sum_{b,i}\sum_k\mathbb{I}(c_{bi}=k)\Big\{-\frac{1}{2}\operatorname{tr}\big[(\mathbf{L}^\top\mathbf{T}_b\mathbf{L}+\omega_{bi}\boldsymbol{\Lambda})\boldsymbol{f}_{bi}\boldsymbol{f}_{bi}^\top\big]+\big[(\boldsymbol{x}_{bi}-\boldsymbol{\gamma}_b)^\top\mathbf{T}_b\mathbf{L}+\omega_{bi}\boldsymbol{\mu}_k^\top\boldsymbol{\Lambda}\big]\boldsymbol{f}_{bi}\\
&\qquad+\frac{1}{2}\log|\mathbf{T}_b|+\frac{d}{2}\log|\omega_{bi}|+\frac{1}{2}\log|\boldsymbol{\Lambda}|-\frac{1}{2}(\boldsymbol{x}_{bi}-\boldsymbol{\gamma}_b)^\top\mathbf{T}_b(\boldsymbol{x}_{bi}-\boldsymbol{\gamma}_b)\\
&\qquad-\frac{1}{2}\omega_{bi}\boldsymbol{\mu}_k^\top\boldsymbol{\Lambda}\boldsymbol{\mu}_k+\eta_b\sum_{j\in\mathsf{N}_{bi}}\mathbb{I}(\hat{c}_j=k)\Big\}+C,\\
Q_2=&\log\big[\mathrm{P}\big(\mathbf{L},\boldsymbol{s}\,|\,\boldsymbol{p}\big)\big]=\log\left[\prod_{i,j}\mathrm{P}\big(L_{ij},s_{ij}\,|\,p_j\big)\right]\\
=&\sum_{i,j}\log\left[\prod_{s\in\{0,1\}}\Big[\mathrm{P}\big(s_{ij}=s\,|\,p_j\big)\mathrm{P}\big(L_{ij}\,|\,s_{ij}=s\big)\Big]^{\mathbb{I}(s_{ij}=s)}\right]\\
=&\sum_{i,j}\Big\{\mathbb{I}(s_{ij}=1)\Big[\log p_j+\log\mathrm{P}\big(L_{ij}\,|\,s_{ij}=1\big)\Big]+\mathbb{I}(s_{ij}=0)\Big[\log(1-p_j)+\log\mathrm{P}\big(L_{ij}\,|\,s_{ij}=0\big)\Big]\Big\}\\
=&\sum_{i,j}\Big\{\mathbb{I}(s_{ij}=1)\Big[\log p_j+\log\frac{L_{ij}^2}{\lambda_1}-\frac{L_{ij}^2}{2\lambda_1}\Big]+\mathbb{I}(s_{ij}=0)\Big[\log(1-p_j)-\frac{L_{ij}^2}{2\lambda_0}\Big]\Big\}+C,\\
Q_3=&\log\left[\pi(\boldsymbol{\gamma})\pi(\boldsymbol{\mu})\pi(\boldsymbol{\omega})\pi(\boldsymbol{p})\pi(\mathbf{T})\pi(\boldsymbol{\Lambda})\right]
\end{aligned}
$$

$$
\begin{aligned}
&= \sum_b \Big(-\frac{1}{2}\|\gamma_b\|_2^2\Big) + \sum_k \Big[-\frac{1}{2}(\boldsymbol{\mu}_k - \boldsymbol{\mu}_\mu)^\top \boldsymbol{\Sigma}_\mu^{-1}(\boldsymbol{\mu}_k - \boldsymbol{\mu}_\mu)\Big] + \sum_{b,i}\Big[\Big(\frac{\nu_\omega}{2} - 1\Big)\log \omega_{bi} - \frac{\nu_\omega}{2}\omega_{bi}\Big] \\
&\quad + \sum_b \Big[\Big(\frac{\nu_\tau}{2} - 1\Big)\log|\mathbf{T}_b| - \frac{\nu_\tau}{2}\operatorname{tr}(\mathbf{T}_b)\Big] + \Big[\frac{n_\Lambda - d - 1}{2}\log|\boldsymbol{\Lambda}| - \frac{1}{2}\operatorname{tr}\big(\boldsymbol{\Sigma}_\Lambda^{-1}\boldsymbol{\Lambda}\big)\Big] \\
&\quad + \sum_j \Big[\Big(\frac{\alpha_p}{j} - 1\Big)\log p_j + (\beta_p - 1)\log(1 - p_j)\Big].
\end{aligned}
$$

Calculation of Eq. (0.4) Substituting Eq. (0.2) into Eq. (0.3) gives Eq. (0.4).

$$
\begin{aligned}
\mathbb{E}_{\tilde{P}_1}[\tilde{Q}_1] = &\sum_{b,i}\sum_k S_{bik}\Big\{\frac{1}{2}\log|\mathbf{T}_b| + \frac{d}{2}\log|\omega_{bi}| + \frac{1}{2}\log|\boldsymbol{\Lambda}| - \frac{1}{2}(\boldsymbol{x}_{bi} - \boldsymbol{\gamma}_b)^\top \mathbf{T}_b(\boldsymbol{x}_{bi} - \boldsymbol{\gamma}_b) \\
&-\frac{1}{2}\boldsymbol{\mu}_k^\top \omega_{bi}\boldsymbol{\Lambda}\boldsymbol{\mu}_k + \eta_b \sum_{j \in \mathsf{N}_{bi}} \mathbb{I}(\hat{c}_j = k) - \frac{1}{2}\operatorname{tr}\Big[(\mathbf{L}^\top \mathbf{T}_b \mathbf{L} + \omega_{bi}\boldsymbol{\Lambda})\mathbb{E}_{P_{11}\,|\,c_{bi}=k}\Big(\boldsymbol{f}_{bi}\boldsymbol{f}_{bi}^\top\Big)\Big] \\
&+\Big[(\boldsymbol{x}_{bi} - \boldsymbol{\gamma}_b)^\top \mathbf{T}_b \mathbf{L} + \boldsymbol{\mu}_k^\top \omega_{bi}\boldsymbol{\Lambda}\Big]\mathbb{E}_{P_{11}\,|\,c_{bi}=k}\Big(\boldsymbol{f}_{bi}\Big)\Big\} + C, \\
\mathbb{E}_{P_2}[Q_2] = &\sum_{i,j}\Big\{h_{ij}\Big[\log p_j + \log\frac{L_{ij}^2}{\lambda_1} - \frac{L_{ij}^2}{2\lambda_1}\Big] + (1 - h_{ij})\Big[\log(1 - p_j) - \frac{L_{ij}^2}{2\lambda_0}\Big]\Big\} + C.
\end{aligned}
$$

All expectations used in calculation of Eq. (0.4) are

$$
\begin{aligned}
\mathbb{E}_{P_2}[\mathbb{I}(s_{ij} = 1)] &= h_{ij}, \\
\mathbb{E}_{P_{11}\,|\,c_{bi}=k}[\boldsymbol{f}_{bi}] &\overset{\text{def}}{=} \mathbb{E}_{bik}[\boldsymbol{f}_{bi}] = \Phi_b^{-1}\varphi_{bik}, \\
\mathbb{E}_{P_{11}\,|\,c_{bi}=k}\Big[\boldsymbol{f}_{bi}\boldsymbol{f}_{bi}^\top\Big] &\overset{\text{def}}{=} \mathbb{E}_{bik}\Big[\boldsymbol{f}_{bi}\boldsymbol{f}_{bi}^\top\Big] = \Phi_{bi}^{-1} + \Phi_{bi}^{-1}\varphi_{bik}\varphi_{bik}^\top\Phi_{bi}^{-1}.
\end{aligned}
$$

And updating subtype indicators $\boldsymbol{c}$ uses

$$
\hat{c}_{bi}^{(t+1)} = \arg\max_k S_{bik} = \arg\max_k \exp\Big[-\frac{1}{2}(\tilde{\boldsymbol{x}}_{bi} - \mathbf{L}\boldsymbol{\mu}_k)^\top \Psi_{bi}(\tilde{\boldsymbol{x}}_{bi} - \mathbf{L}\boldsymbol{\mu}_k) + \eta_b \sum_{j \in \mathsf{N}_{bi}} \mathbb{I}(\hat{c}_j = k)\Big].
$$

Calculation of Eq. (0.5) To obtain Eq. (0.5), we will derive the partial derivative of each variable under all the others fixed, then obtain the following

$$
\begin{aligned}
\{\mathbf{T}_b^{-1}\}^{(t+1)} = &\frac{1}{n_b + \nu_\tau - 2}\text{diag}\Big\{\sum_{i \in \mathsf{B}_b}\Big[\tilde{\boldsymbol{x}}_{bi}\tilde{\boldsymbol{x}}_{bi}^\top + \mathbf{L}\Big(\sum_k S_{bik}\mathbb{E}_{bik}\Big[\boldsymbol{f}_{bi}\boldsymbol{f}_{bi}{}^\top\Big]\Big)\mathbf{L}^\top \\
&-\tilde{\boldsymbol{x}}_{bi}\Big(\sum_k S_{bik}\mathbb{E}_{bik}[\boldsymbol{f}_{bi}]\Big)^\top \mathbf{L}^\top - \mathbf{L}\Big(\sum_k S_{bik}\mathbb{E}_{bik}[\boldsymbol{f}_{bi}]\Big)\tilde{\boldsymbol{x}}_{bi}^\top\Big] + \nu_\tau \mathbf{I}_p\Big\}, \\
\boldsymbol{\gamma}_b^{(t+1)} = &(n_b\mathbf{T}_b + \mathbf{I}_p)^{-1}\sum_{i \in \mathsf{B}_b}\mathbf{T}_b\Big(\boldsymbol{x}_{bi} - \mathbf{L}\sum_k S_{bik}\mathbb{E}_{bik}[\boldsymbol{f}_{bi}]\Big), \\
\boldsymbol{\mu}_k^{(t+1)} = &\Big(\sum_{b,i} S_{bik}\omega_{bi}\boldsymbol{\Lambda} + \boldsymbol{\Sigma}_\mu^{-1}\Big)^{-1}\Big(\sum_{b,i} S_{bik}\omega_{bi}\boldsymbol{\Lambda}\mathbb{E}_{bik}[\boldsymbol{f}_{bi}] + \boldsymbol{\Sigma}_\mu^{-1}\boldsymbol{\mu}_\mu\Big),
\end{aligned}
$$

$$\omega_{bi}^{(t+1)} = \frac{d+\nu_\omega-2}{\sum_k S_{bik}\Big(\boldsymbol{\mu}_k^\top\boldsymbol{\Lambda}\boldsymbol{\mu}_k + \mathrm{tr}\big(\boldsymbol{\Lambda}\mathbb{E}_{bik}\left[\boldsymbol{f}_{bi}\boldsymbol{f}_{bi}{}^\top\right]\big) - 2\boldsymbol{\mu}_k^\top\boldsymbol{\Lambda}\mathbb{E}_{bik}[\boldsymbol{f}_{bi}]\Big) + \nu_\omega},$$

$$\{\boldsymbol{\Lambda}^{-1}\}^{(t+1)} = \frac{1}{n+n_\Lambda-d-1}\left\{\sum_{b,i,k}\omega_{bi}S_{bik}\Big(\mathbb{E}_{bik}\left[\boldsymbol{f}_{bi}\boldsymbol{f}_{bi}{}^\top\right] + \boldsymbol{\mu}_k\boldsymbol{\mu}_k^\top - \boldsymbol{\mu}_k\mathbb{E}_{bik}[\boldsymbol{f}_{bi}]^\top - \mathbb{E}_{bik}[\boldsymbol{f}_{bi}]\,\boldsymbol{\mu}^\top\Big) + \boldsymbol{\Sigma}_\Lambda^{-1}\right\},$$

$$p_j^{(t+1)} = \frac{\sum_i h_{ij} + \alpha_{p_j}/j - 1}{p + \alpha_{p_j} + \beta_p - 2},$$

$$\frac{\partial\widetilde{Q}(\theta \mid \theta^{(t)})}{\partial L_{ij}} = \frac{1}{L_{ij}}\Bigg\{\underbrace{-\left[\frac{h_{ij}}{\lambda_1} + \frac{1-h_{ij}}{\lambda_0} + \sum_{b,s}\tau_{bi}\Big(\sum_k S_{bsk}\mathbb{E}_{bik}\left[f_{bsj}{}^2\right]\Big)\right]}_{a_{ij}} L_{ij}^2 + \underbrace{2h_{ij}}_{c_{ij}}$$

$$+ \underbrace{\sum_{b,s}\left[\tau_{bi}\tilde{\boldsymbol{x}}_{bsi}\Big(\sum_k S_{bsk}\mathbb{E}_{bik}[f_{bsj}]\Big) - \sum_{u\neq j}\tau_{bi}L_{iu}\sum_k S_{bsk}\Big(\mathbb{E}_{bik}\big(f_{bsu}f_{bsj}\big)\Big)\right]}_{b_{ij}} L_{ij}\Bigg\}.$$

Define

$$\overline{L}_{ij} = \frac{-b_{ij} + \sqrt{b_{ij}^2 - 4a_{ij}c_{ij}}}{2a_{ij}}, \qquad \underline{L}_{ij} = \frac{-b_{ij} - \sqrt{b_{ij}^2 - 4a_{ij}c_{ij}}}{2a_{ij}},$$

then from Lemma 1 in [Avalos-Pacheco et al., 2022], we have

$$\widehat{L}_{ij} = \mathbb{I}(b_{ij} < 0)\,\overline{L}_{ij} + \mathbb{I}(b_{ij} > 0)\,\underline{L}_{ij}.$$

To make our paper self-contained, the lemma is given below.

Lemma 0.2 ([Avalos-Pacheco et al., 2022]). *Let* $f(x) = ax^2 + bx + c\log(x^2)$*, where* $a < 0$ *and* $c > 0$*, with notation* $\Delta = b^2 - 16ac$*, we have*

$$\widehat{x} = \arg\max_x f(x) = \begin{cases} 0 & \text{if } \Delta < 0, \\ \frac{-b+\sqrt{\Delta}}{4a} & \text{if } \Delta \geqslant 0 \text{ and } b < 0, \\ \frac{-b-\sqrt{\Delta}}{4a} & \text{if } \Delta \geqslant 0 \text{ and } b > 0, \\ \pm\sqrt{\frac{-c}{a}} & \text{if } \Delta \geqslant 0 \text{ and } b > 0. \end{cases}$$

Proof. Taking derivative of $f(x)$ with respect to x is

$$\frac{\mathrm{d}f(x)}{\mathrm{x}} = 2ax + b + \frac{2c}{x} = \frac{1}{x}\left(2ax^2 + bx + 2c\right),$$

with corresponding discriminant denoted by

$$\Delta = b^2 - 16ac \geqslant 0,$$

wherein Δ being always positive is from $a < 0$ and $c > 0$. Then roots formula of quadratic equation gives

$$\bar{x} = \frac{-b+\sqrt{\Delta}}{4a} \leqslant 0, \qquad \underline{x} = \frac{-b-\sqrt{\Delta}}{4a} \geqslant 0.$$

To check which of $\bar{x}$ and $\underline{x}$ is maximizer, their functional values are compared

$$f(\bar{x}) - f(\underline{x}) = \frac{b\sqrt{\Delta}}{4a} + c\log\left[\left(\frac{-b+\sqrt{\Delta}}{-b-\sqrt{\Delta}}\right)^2\right].$$

- If $b > 0$, with $a < 0$, we have $b\sqrt{\Delta}/a < 0$ and $| -b + \sqrt{\Delta} | < | -b - \sqrt{\Delta} |$, then $f(\bar{x}) < f(\underline{x})$.
- If $b < 0$, with $a < 0$, we have $b\sqrt{\Delta}/a > 0$ and $| -b + \sqrt{\Delta} | > | -b - \sqrt{\Delta} |$, then $f(\bar{x}) > f(\underline{x})$.
- If $b = 0$, we have $b\sqrt{\Delta}/a = 0$ and $| -b + \sqrt{\Delta} | = | -b - \sqrt{\Delta} |$, then $f(\bar{x}) = f(\underline{x})$.

□

Compared methods

We compared STADIA with three competing data integration methods, including PRECAST, fastMNN, and Harmony. For fairness of comparison, all methods adopt the same data preprocessing procedure, described in the main text. And the implementation of all compared methods were followed the authors' instructions.

- **PRECAST[Liu et al., 2023].** First `GetAssayData` and *$* functions from the R package Seurat are used to extract gene expression profiles and coordinates for all spots. Then `ICM.EM` function from the R package PRECAST (v1.6) with low dimensionality parameter q equal to 35. Finally select the final result using the function `selectModel` in the R package PRECAST.

- **fastMNN[Haghverdi et al., 2018].** FastMNN is a fast version of MNN following principal component analysis (PCA). Based on the selected HVGs, we used the `RunFastMNN` function in the R package SeuratWrappers (v0.3.1), adapting `fastMNN` in the R package batchelor, with the top 35 PCs and all other parameters by default.

- **Harmony[Korsunsky et al., 2019].** First get the top 35 PCs with the function `RunPCA` in the R package Seurat (v4.3.0) and then do Harmony with the function `RunHarmony` in the R package harmony (v0.1.1) with all default parameters.

- **STAGATE[Dong and Zhang, 2022]** STAGATE first builds a Spatial Neighbor Network (SNN) using a predetermined radius, and then uses a four-layer graph attention autoencoder to learn low-dimensional embeddings. We conducted the experiments according to the provided tutorial https://stagate.readthedocs.io/en/latest/AT2.html.

- **GraphST[Long et al., 2023].** GraphST is a graph self-supervised contrastive learning method designed to facilitate spatial clustering and multi-slice integration. We conducted the experiments according to the provided tutorial https://deepst-tutorials.readthedocs.io/en/latest/Tutorial%201_10X%20Visium.html.

- **SpatialDE[Svensson et al., 2018].** SpatialDE identifies spatial variable genes by modeling their dependence on spatial coordinates using non-linear and non-parametric Gaussian process regression. We conducted the experiments according to the provided tutorial https://github.com/Teichlab/SpatialDE.

- **SPARK-X[Zhu et al., 2021].** SPARK-X is a scalable, non-parametric method that employs a robust covariance test framework to model a wide variety of spatial transcriptomics datasets. We conducted the experiments according to the provided tutorial https://xzhoulab.github.io/SPARK/02_SPARK_Example/.

Evaluation metric

- **Adjusted Rand Index (ARI)[Hubert and Arabie, 1985]**, adapting the Rand Index (RI), measures the similarity between two partitions, defined as

$$\text{ARI} = \frac{\sum_{ij}\binom{n_{ij}}{2} - \left[\sum_i \binom{a_i}{2}\sum_j \binom{b_j}{2}\right] / \binom{n}{2}}{\frac{1}{2}\left[\sum_i \binom{a_i}{2} + \sum_j \binom{b_j}{2}\right] - \left[\sum_i \binom{a_i}{2} + \sum_j \binom{b_j}{2}\right] \binom{n}{2}},$$

  where a and b are two partitions, n is the total number of nodes, a_i and b_j are the number of nodes in particular partitions, and n_{ij} is the number of cells that appear simultaneously in the ith cluster of partition a and the jth cluster of partition b. ARI is a symmetric measure that ranges from 0 to 1, and higher value indicates higher similarity. We compute ARI to compare the clustering result of the integrated data with the predefined cell types.

- **Normalized Mutual Information (NMI)[Shannon, 1948]**, is the normalization of the Mutual Information (MI) used to measure clustering accuracy. NMI is defined as

$$\text{NMI} = 2 \times \frac{\sum_{ij} \frac{n_{ij}}{n} \log\left(\frac{n \times n_{ij}}{a_i \times b_j}\right)}{\sum_i \frac{a_i}{n} \log\left(\frac{n}{a_i}\right) + \sum_j \frac{b_j}{n} \log\left(\frac{n}{b_j}\right)},$$

  where the notation is the same as that in ARI. NMI ranges in $[0, 1]$ and higher value also indicates higher similarity between the clustering result and the true cell types.

- **Local Inverse Simpson's Index of Integration (LISI)[Korsunsky et al., 2019]** is used to evaluate the quality of mixing after integration. Based on local neighbors chosen on a preselected perplexity, LISI calculates the effective number of different batches in the local neighborhood of each cell for *iLISI* score, and of different cell types for *cLISI* score. From a perspective of application, the higher value of iLISI and lower value of cLISI mean better mixing.

Data preprocessing

We first performed quality control on all datasets (see **Supplementary Table S1** for details) by filtering out spots expressed in less than 200 genes and genes expressed in less than

20 spots. Then, in each experiment, we selected the top 2000 highly variable genes (HVGs) (default) or the top 2000 spatial variable genes (SVGs) detected by SpatialDE[Svensson et al., 2018], SPARK-X[Zhu et al., 2021] or other methods per slice, ranked them by the number of slices they appear in, and took the top 2000 features as input. Finally, we normalized the raw count data according to the library size and performed a log transformation. We implemented these steps by the Seurat package (v4.3.0) [Hao et al., 2021], including the functions `FindVariableFeatures`, `SelectIntegrationFeatures`, `NormalizeData`, and `ScaleData`.

Differential expression analysis and GO enrichment analysis

For differential expression analysis, we used the `FindAllMarkers` function in the R package Seurat (v4.3.0) to perform the Wilcoxon rank-sum test with gene expression at least a $0.25-$fold difference (log-scale) between the target domain and others. We obtained the final DEGs for each domain using the adjusted p-value (Benjamin-Hochberg correction) with a cutoff of 0.05.

For GO enrichment analysis, we first used the `select` function in the R package AnnotationDbi (v1.60.2) to transfer the gene symbol to entrezid. For the DEGs of each domain, we used the `enrichGO` function in the R package clusterProfiler (v4.6.2) to perform GO enrichment analysis for gene ontology over-representation test (one-sided version of Fisher's exact test).

Data availability

All datasets analyzed in this study are available through websites reported in the original publications. Specifically, the Human dorsolateral prefrontal cortex (DLPFC) data can be accessed in the spatialLIBD package, which is available at http://research.libd.org/spatialLIBD/. The mouse sagittal posterior and anterior brain data can be accessed in official 10x genomics support https://support.10xgenomics.com/spatial-gene-expression/datasets/1.0.0/V1_Mouse_Brain_Sagittal_Anterior, https://support.10xgenomics.com/spatial-gene-expression/datasets/1.0.0/V1_Mouse_Brain_Sagittal_Posterior. The cutaneous squamous cell carcinoma (cSCC) data can be accessed at the Gene Expression Omnibus (GEO) under the accession code GSE144240. The mouse liver data are available in the DOI-minting Zenodo repository 5595907. The mouse hippocampus dataset can be accessed at https://singlecell.broadinstitute.org/single_cell/study/SCP815 . The image of Allen Mouse Brain Atlas can be accessed at http://atlas.brain-map.org/atlas?atlas=2&plate=100883818.

Further Analysis

STADIA learns the common biological variations among eight mouse liver tissue sections: We further applied STADIA to the mouse liver dataset [Hildebrandt et al., 2021], which consists of eight liver tissue slices from three adult female wild mice profiled by the ST protocol. Six slices were from the caudate lobe portion of mouse 1 (CN73) and mouse 2 (CN65) (three slices per mouse), and two were from the right lobe portion of mouse 3 (CN16) (**Fig. S5a**).

From the UMAP plot of the uncorrected raw data, there were significant differences between the three mice, with individual CN73 being closer to CN65 than CN16, which may because both CN73 and CN65 were sampled from the caudate lobe (**Fig. S5b, left panel**). Batch effects across slices from the same individual were negligible (**Fig. S3a**). In addition, STADIA mixed all eight slices well and separated the six domains on the embedded UMAP plot (**Fig. S5b, middle and right panels**). Using the Wilcoxon rank-sum test, we found the domain-specific marker genes and plotted the top five for each domain in a dot plot (**Fig. S5c**). The top five marker genes for domain 1 and domain 4 included previously published zoned hepatocyte genes *Glul*, *Cyp2e1*, *Sds*, and *Hal* [Guilliams et al., 2022], suggesting that domain 1 and domain 4 are central vein (CV) and portal vein (PV), respectively. To further confirm our findings, we calculated Pearson correlations of marker genes for domain 1 and domain 4 (**Fig. S5d** and **Fig.S3c**) and visualized their expression in a spatial context (**Fig. S5e**), which indicated that the marker gene expression of these two organizational structures was negatively correlated.

We further explored the biological significance of differentially expressed genes (DEGs) by Gene Ontology (GO) enrichment analysis in terms of biological process (BP), cellular component (CC) and molecular function (MF). Among them, focusing on up-regulated DEGs for domain 2, the enriched biological processes were mainly related to ribonucleoprotein complex assembly and ribonucleoprotein complex biogenesis. In terms of cellular components, DEGs were enriched in ribosomes of organelles and ribosomes. In terms of molecular function, only the terms for structural constituent of ribosome and protein kinase B binding were enriched (**Fig. S5f**). DEGs for other domains were mainly enriched in different metabolic pathways (**Fig. S3d**).

Evaluation with simulated data

To further demonstrate STADIA's ability to handle different scales of batch effect, we conducted simulation studies. To ensure that the spatial coherence is biologically meaningful, we adopted domain labels and spatial locations from three different donor DLPFC slices (151507, 151669, 151573)[Maynard et al., 2021]. We then generated gene expression profiles using a seven-component Gaussian mixture model in two dimensions $p(x) = \sum_{k=1}^{7} \pi_k \mathrm{N}(\mu_\mathrm{k}, \Sigma_\mathrm{k})$, representing the embedded biological subspace. Each μ_k was drawn from a Gaussian distribution with a mean of zero and a standard deviation of 5, and each Σ_k was either an identity matrix or a diagonal matrix with elements drawn from a Gamma(1,1) distribution. The data from all three batches were projected into G=2000 dimensions using the same random Gaussian matrix to preserve the similar biological variation. Finally, batch effects were introduced by generating Gaussian random vectors $\{\gamma_b : b = 1, 2, 3\}$, where each element of $\gamma_b \sim \mathrm{N}(0, \omega_{\text{scale}} * \nu_\mathrm{b})$ with $\nu_1 = 0, \nu_2 = 0.5, \nu_3 = 1$. The parameter $\omega_{\text{scale}} \in \{0.5, 1, 2\}$ was used to control the scale of batch effects.

The three rows in **Fig. S8** represent small, medium and large batch effects. In the small batch effect scenario, only the third batch (yellow) exhibits slight batch effects compared to the other two batches. In the medium scenario, the yellow batch shows more pronounced batch effects compared to the green and purple batches. And in the large scenario, all batches are

distinctly separated.

We applied STADIA to the simulated data sets. As shown in the second and third columns of **Fig. S8a, b**, STADIA effectively removed the batch effects across three different scales in both two scenarios, uncovering the underlying biological variations. Comparing **Fig. S8a** and **Fig. S8b**, STADIA effectively demonstrated its ability to remove batch effects across data sets with different distributions. The UMAP plots for STADIA are similar across each scenario because, despite the batch effects being scaled differently, they were all added to the same underlying biological data.

Supplementary Figures

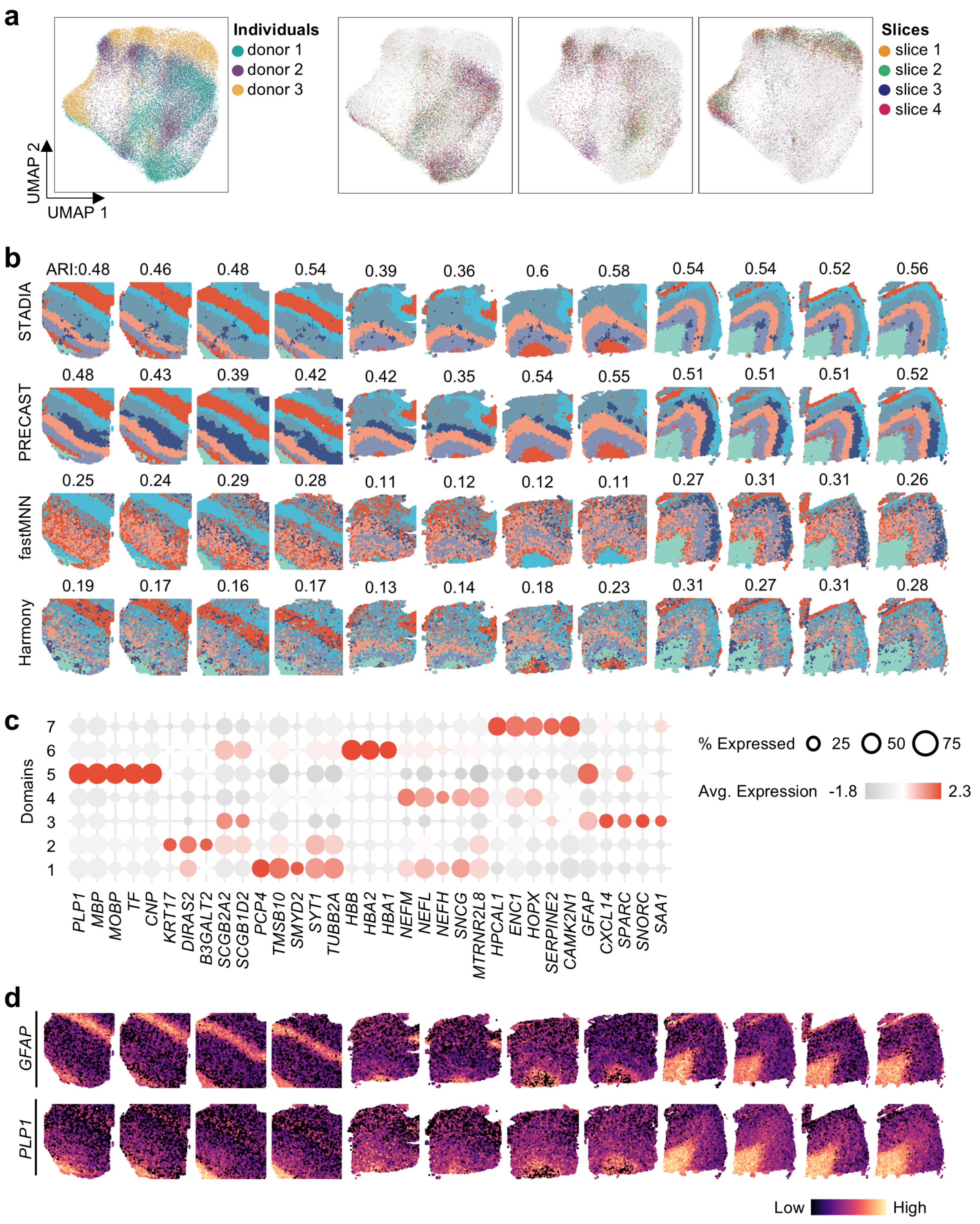

Fig. S1. Integration of 12 slices of DLPFC dataset. **a.** Embedded UMAP plots for original raw data colored by different donors (left) and slices per donor (left). **b.** Visualization of the spatial domains for the four methods with the corresponding ARI marked on each slice. **c.** Pearson correlations between the domains identified by STADIA. **d.** Dot plot of the top 5 marker genes for each domain identified by STADIA. **e.** Visualization of the expression of markers for layer 1 and WM in spatial context.

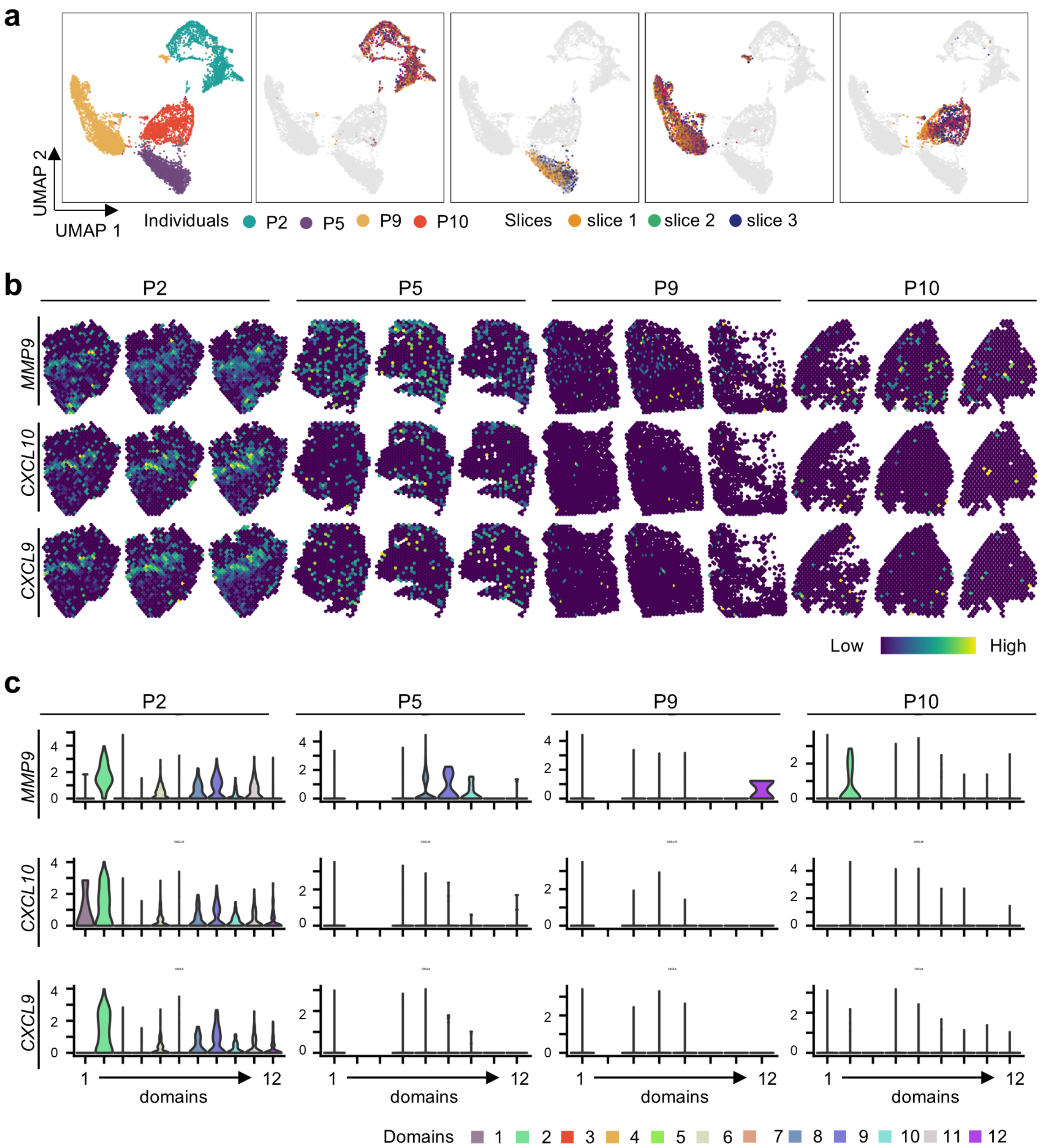

Fig. S2. Integration of 12 slices of cSCC dataset. a. Embedded UMAP plots for original raw data colored by different patients (left) and slices per patient (left). **b.** Visualization of the expression of markers *MMP9*, *CXCL10*, *CXCL9* for domain 2 in spatial context. **c.** Violin plot of the expression of genes *MMP9*, *CXCL10*, *CXCL9*.

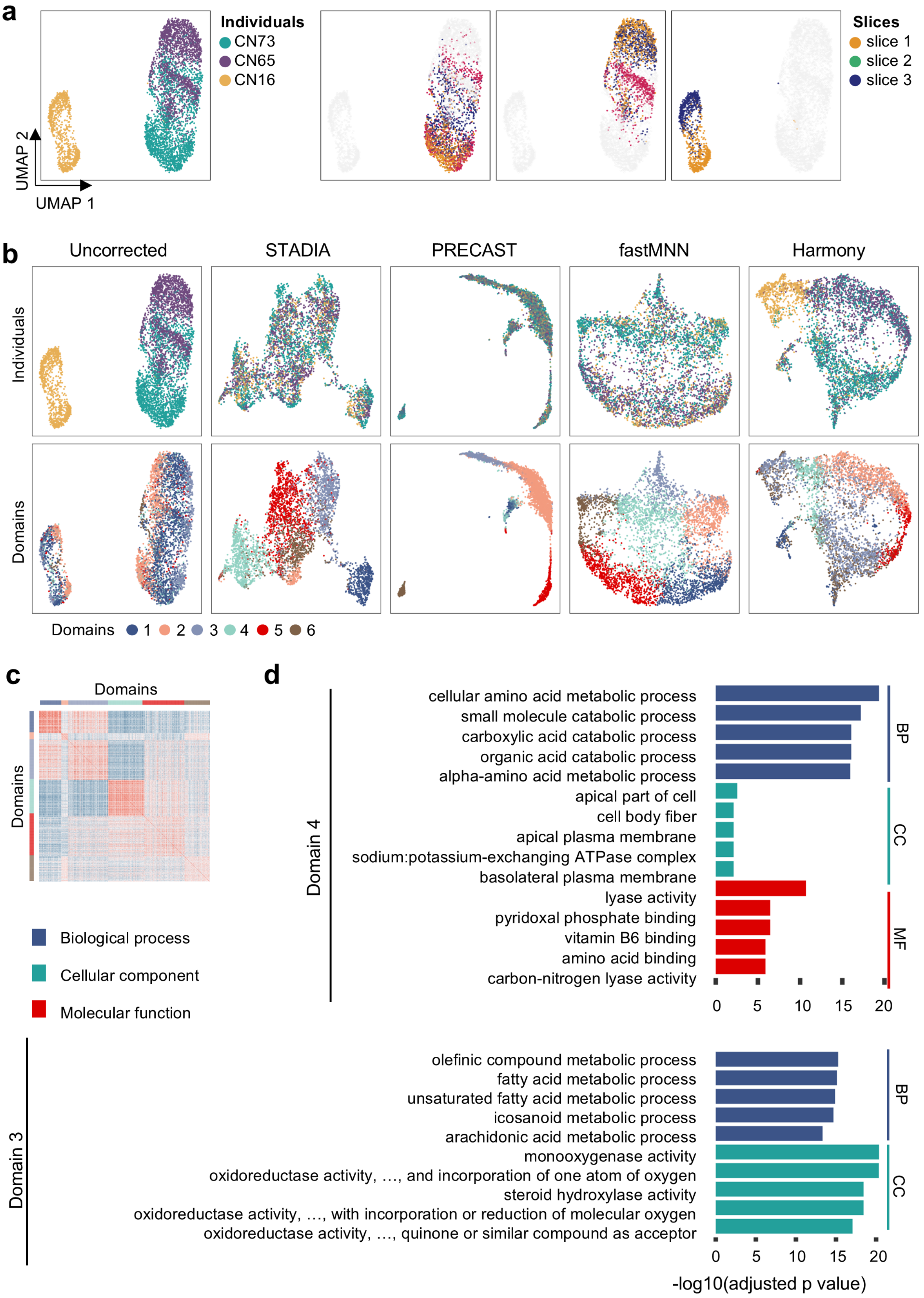
a
Individuals
CN73
CN65
CN16
UMAP 2
UMAP 1
Slices
slice 1
slice 2
slice 3
b
Uncorrected
STADIA
PRECAST
fastMNN
Harmony
Individuals
Domains
Domains 1 2 3 4 5 6
c
Domains
Domains
Biological process
Cellular component
Molecular function
d
Domain 4
cellular amino acid metabolic process
small molecule catabolic process
carboxylic acid catabolic process
organic acid catabolic process
alpha-amino acid metabolic process
apical part of cell
cell body fiber
apical plasma membrane
sodium:potassium-exchanging ATPase complex
basolateral plasma membrane
lyase activity
pyridoxal phosphate binding
vitamin B6 binding
amino acid binding
carbon-nitrogen lyase activity
BP
CC
MF
0 5 10 15 20
Domain 3
olefinic compound metabolic process
fatty acid metabolic process
unsaturated fatty acid metabolic process
icosanoid metabolic process
arachidonic acid metabolic process
monooxygenase activity
oxidoreductase activity, …, and incorporation of one atom of oxygen
steroid hydroxylase activity
oxidoreductase activity, …, with incorporation or reduction of molecular oxygen
oxidoreductase activity, …, quinone or similar compound as acceptor
BP
CC
0 5 10 15 20
-log10(adjusted p value)

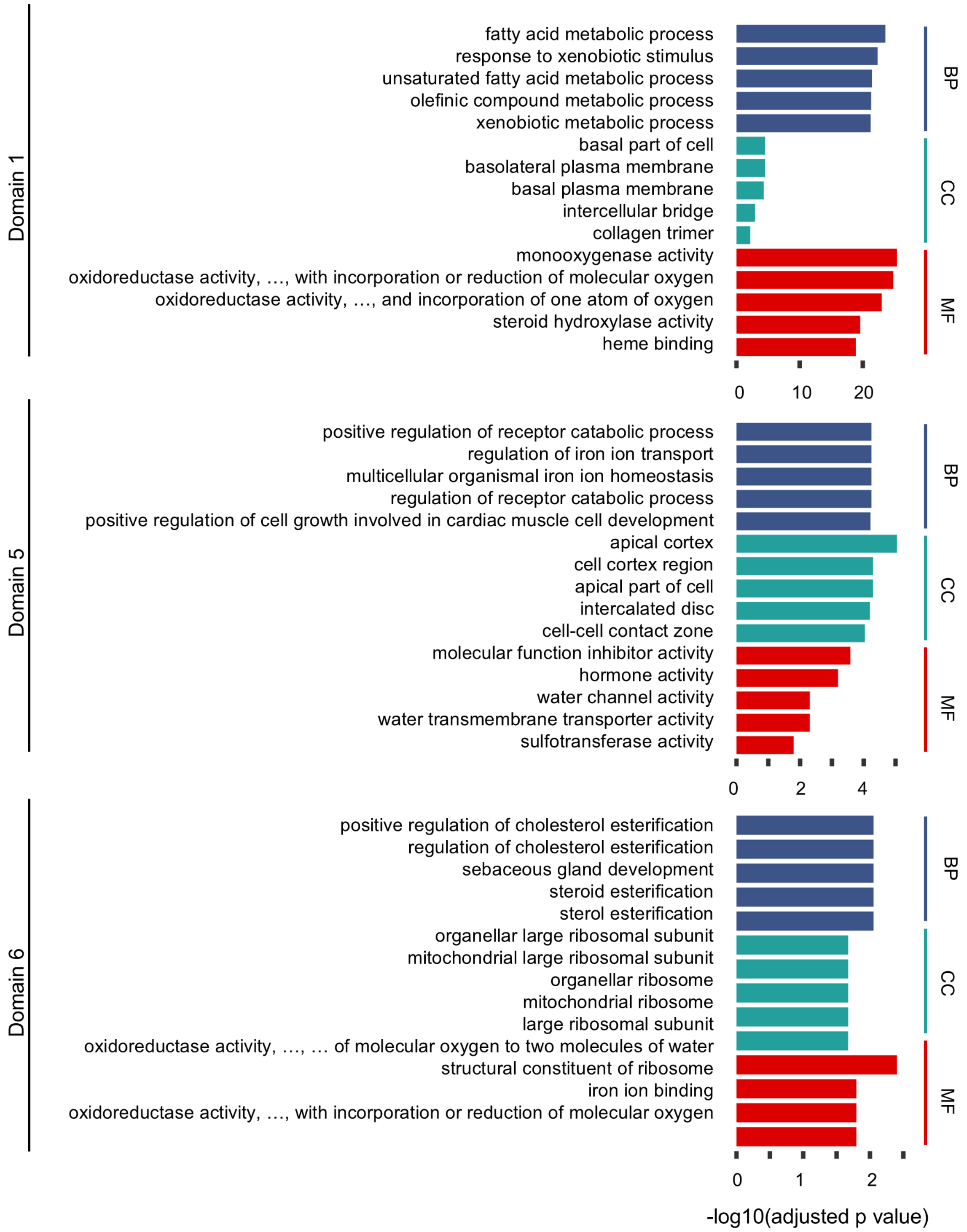

Fig. S3. Integration of 12 slices of cSCC dataset. a. Embedded UMAP plots for original raw data colored by different mice (left) and slices per mouse (left). **b.** Embedded UMAP plots for the four methods colored by mice (top) and spatial domains (bottom). **c.** Pearson correlations between the domains identified by STADIA. **d.** The top 5 most significant GO terms for all domains except for doamin 2, which is also in **Fig. 4**.

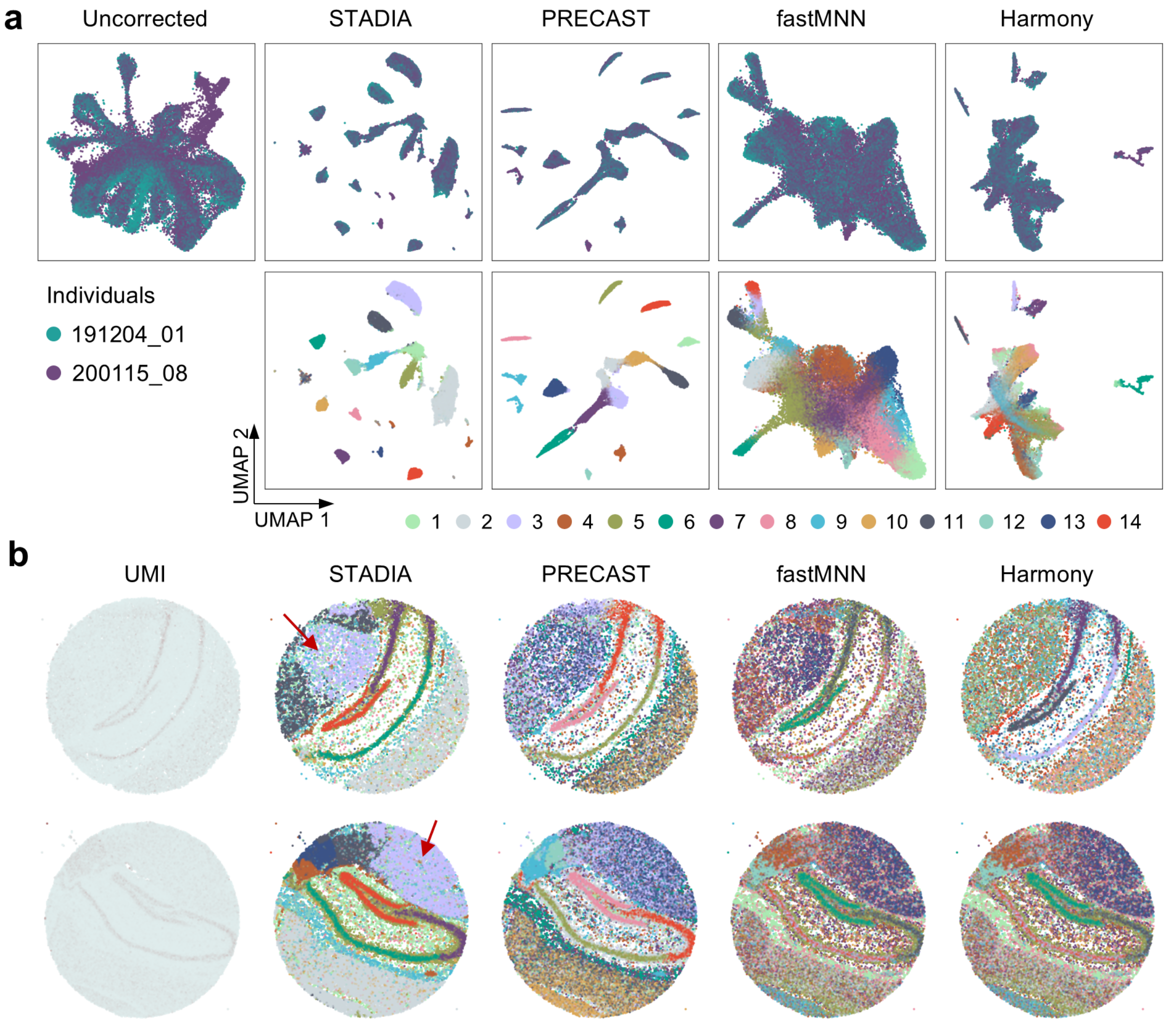

Fig. S4. Integration of two slices of the hippocampal dataset profiled by slide-seqV2. a. Embedded UMAP plots for the four methods colored by slices (top) and spatial domains (bottom). **b.** Visualization of spatial domains identified by the four methods in spatial context.

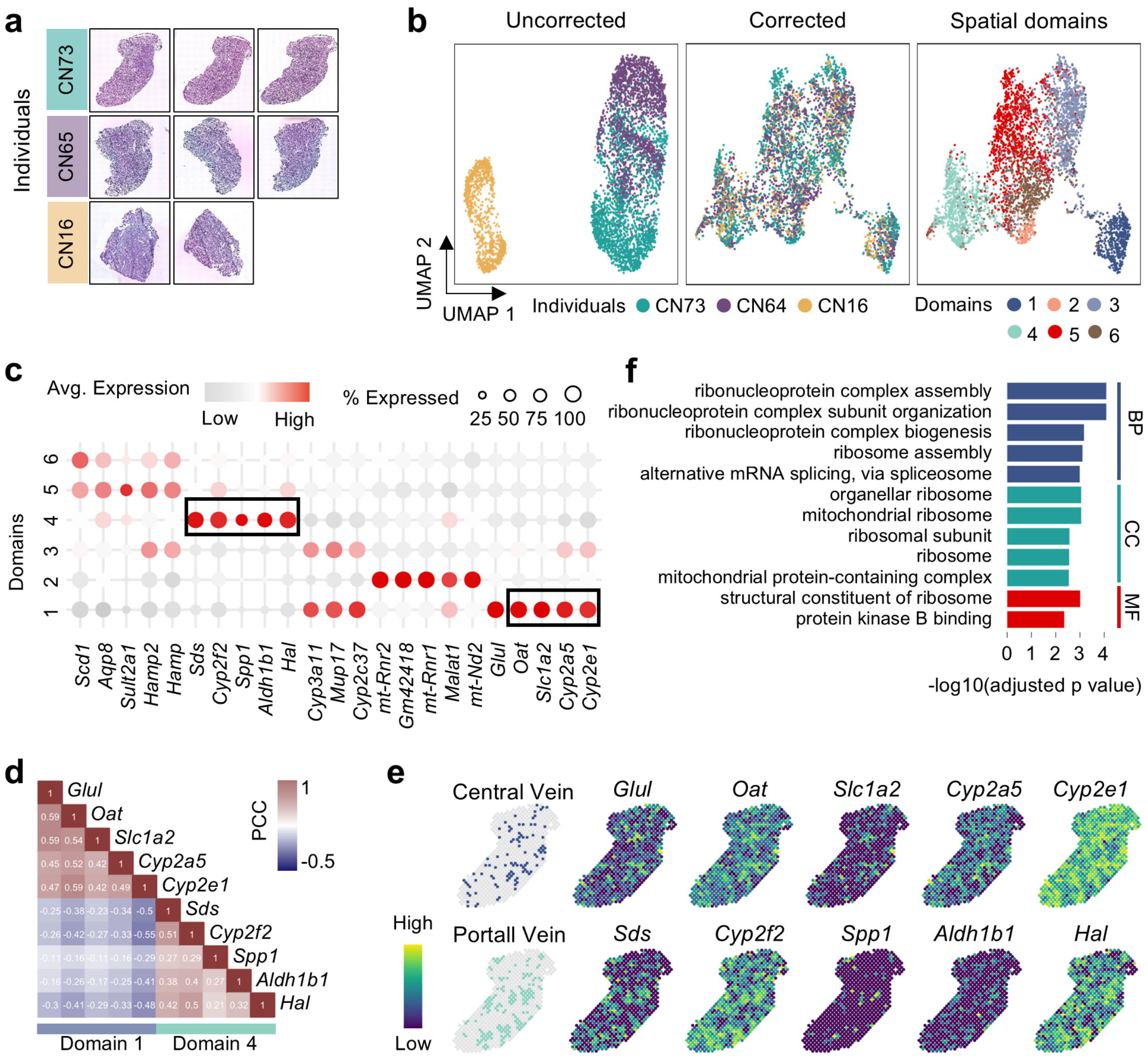

Fig. S5. STADIA learns the common biological variations among eight mouse liver tissue sections. **a**. Hematoxylin and Eosin (H&E) images for all eight mouse liver tissue sections. Slices from the first two samples (CN73, CN65) are from parts of the caudate lobe, and slices from the last sample (CN16) are from parts of the right lobe. **b**. UMAP plots of the original data without correction, colored by mouse (left panel), UMAP plots of embeddings for STADIA, colored by mouse (middle panel), and cluster assignment (right panel). **c**. Dot plot of top five found by the Wilcoxon rank-sum test for each spatial domain identified by STADIA. **d**. Heatmap of Pearson's correlation of the marker gene expression profiles for domains 1 and 4. **e**. Spatial distribution of the marker genes for spatial domains 1 (central vein) and 4 (portal vein) as listed in (c) and (d). **f**. Bar chart of the GO enrichment analysis results for spatial domain 2. The enrichment score is calculated as the -log10(adjusted p-value).

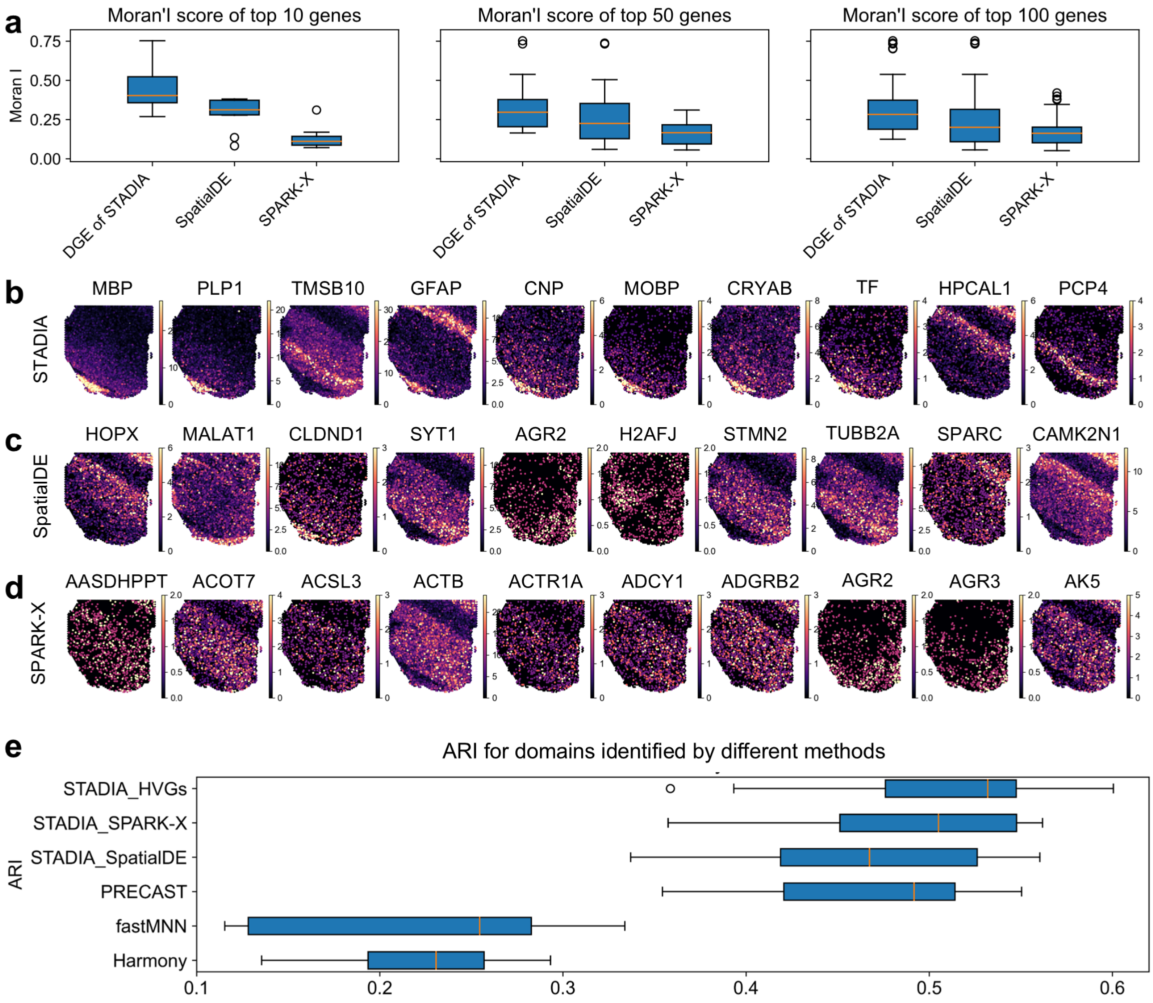

Fig. S6. Evaluating SVGs detecting by STADIA and accessing the robustness of STADIA with respect to the gene sets using as inputs. **a**. Moran I scores of the top SVGs that were detected by STADIA, SpatialDE and SPARK-X. In the boxplot, the center line, box limits and whiskers denote the median, upper and lower quartiles and 1.5×interquartile range, respectively. **b**. Visualization of the top 10 SVGs identified by STADIA. **c**. Visualization of the top 10 SVGs identified by SpatialDE. **d**. Visualization of the top 10 SVGs identified by SPARK-X. **e**. Boxplots of clustering accuracy for different methods in terms of ARI.

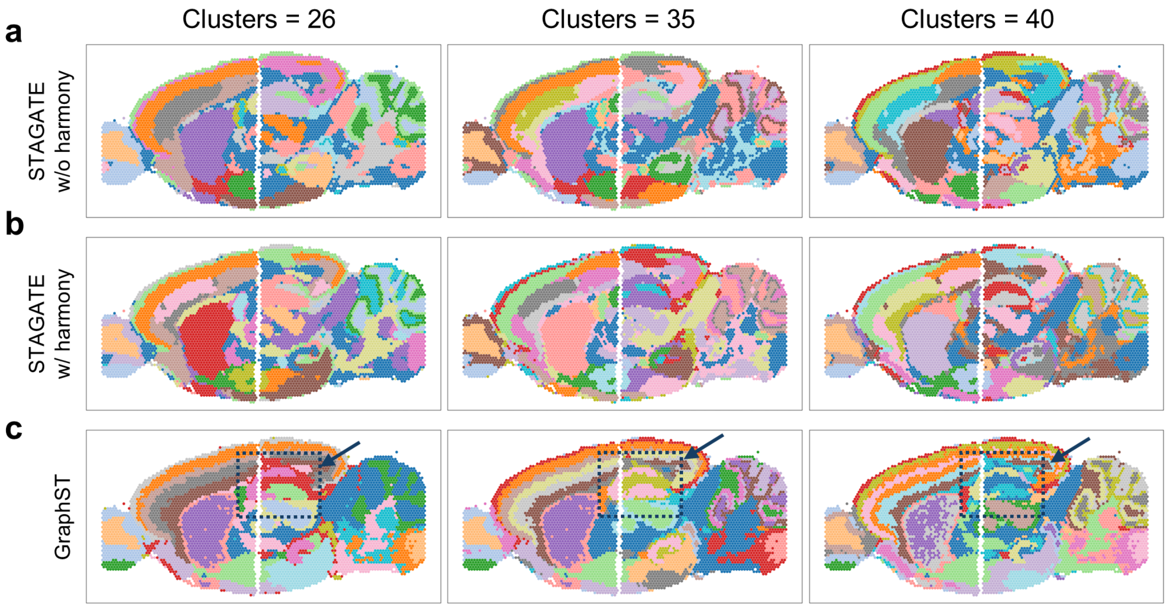

Fig. S7. Alignment of spatial domains identified by STAGATE and GraphST with clusters being 26, 35 and 40, where 26 is the number used in the original publication of GraphST. **a**. Alignment of STAGATE without Harmony. **b**. Alignment of STAGATE with Harmony. **c**. Alignment of GraphST.

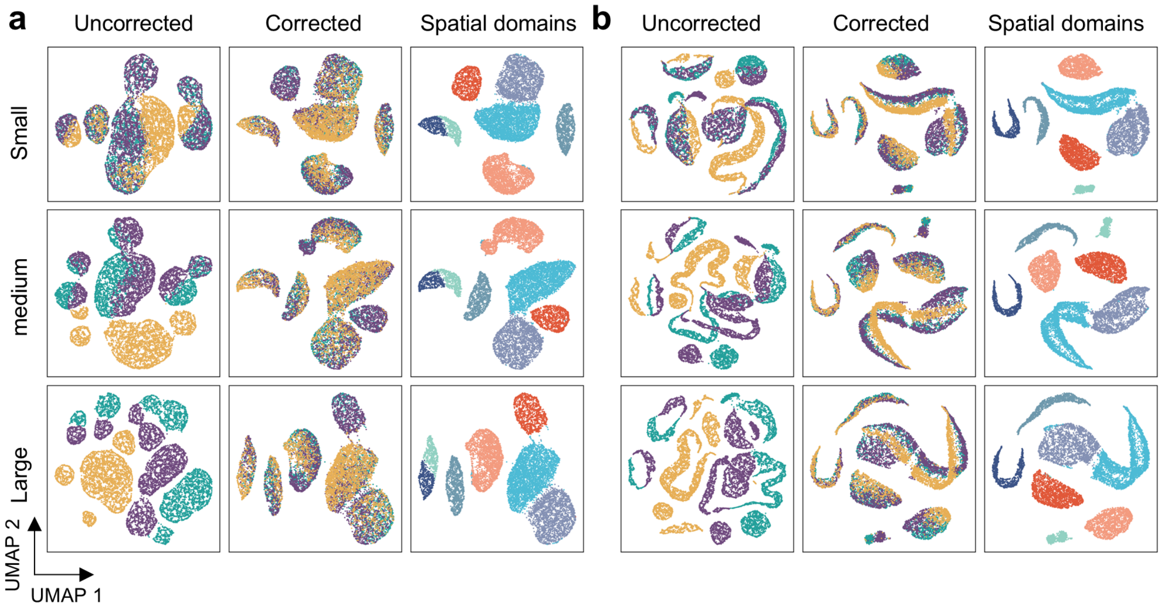

Fig. S8. STADIA removes different scales of batch effects. a and **b**. UMAP plots of the original data without correction colored by batches (left panel), and embeddings for STADIA colored by batches (middle panel) and cluster assignments (right panel), for GMM with diagonal covariance matrices (**a**) and standardized variation generated from a Gamma(1,1) distribution (**b**).

Supplementary Table

Table S1. Summary of all ST data used in this study.

Platform	Species	Tissue	Slices	Spots	Related Figures	Reference
10x Visium	Human	Dorsolateral prefrontal cortex (DLPFC)	151507	4226	Fig. 2<br>Fig. S1	Maynard et al. [2021]
			151508	4384		
			151509	4789		
			151510	4634		
			151669	3661		
			151670	3498		
			151671	4110		
			151672	4015		
			151673	3639		
			151674	3673		
			151675	3592		
			151676	3460		
	Mouse	Brain	Anterior	2696	Fig. 3	10x Visium demo
			Posterior	3353		
ST	Human	Cutaneous squamous cell carcinoma (cSCC)	P2_rep1	666	Fig. 5<br>Fig. S3	Ji et al. [2020]
			P2_rep2	645		
			P2_rep3	638		
			P5_rep1	584		
			P5_rep2	517		
			P5_rep3	517		
			P9_rep1	1125		
			P9_rep2	1035		
			P9_rep3	828		
			P10_rep1	545		
			P10_rep2	619		
			P10_rep3	460		
	Mouse	Liver	CN73_C1	673	Fig. 4<br>Fig. S2	Hildebrandt et al. [2021]
			CN73_D1	684		
			CN73_E2	650		
			CN65_D1	663		
			CN65_D2	629		
			CN65_E1	590		
			CN16_D2	487		
			CN16_E2	487		
Slide-seqV2	Mouse	Hippocampus	Puck_ 191204_01	34199	Fig. 6	Stickels et al. [2021]
			Puck_ 200115_08	53208		

Table S2. The Allen Reference Atlas of Mouse Brain.

Related figures	ABA ID	ABA URL
Fig. 3a	100883818	http://atlas.brain-map.org/atlas?atlas=2&plate=100883818
Fig. 6a	100960084	http://atlas.brain-map.org/atlas?atlas=1&plate=100960084

Table S3. Parameters Used in Experiments.

Dataset	d	K	eta
Human dorsolateral prefrontal cortex (DLPFC)	35	7	0.23
Mouse brain	35	35	0.15
Human cutaneous squamous cell carcinoma (cSCC)	35	12	0.15
Mouse liver	35	6	0.15
Mouse Hippocampus	35	14	0.23

Table S4. Summary of the five datasets used in this study.

Datasets	Resolution	Spatial coherence	Organ	Healthy	Species
cSCC	$100\mu m$	low	Skin	No	Human
Liver	$100\mu m$	low	Liver	Yes	Mouse
DLPFC	$55\mu m$	high	Brain	Yes	Human
Sagittal brain	$55\mu m$	high	Brain	Yes	Mouse
Hippocampus	$10\mu m$	high	Brain	Yes	Mouse

Table S5. Computational time and memory usage for all experiments on a machine with a 48-core AMD EPYC 7K62 CPU and an NVIDIA GeForce RTX 4090 GPU.

Datasets	Object size (GB)	NO. of locations	Methods	Computation time (min)	Memory usage (GB)
DLPFC	2.04	47,681	fastMNN	3.03	10.98
			Harmony	1.80	6.78
			PRECAST	41.54	6.46
			STADIA	43.13	104.81
Sagittal Brain	0.71	6,049	fastMNN	0.36	3.24
			Harmony	0.22	2.78
			PRECAST	24.3	2.26
			STADIA	3.33	2.78
cSCC	0.32	8,179	fastMNN	0.60	2.20
			Harmony	0.23	1.83
			PRECAST	4.87	2.36
			STADIA	3.41	3.75
Hippocampus	0.81	87,407	fastMNN	1.38	6.42
			Harmony	1.00	3.33
			PRECAST	35.42	16.99
			STADIA	18.75	88.37
Liver	0.28	4,863	fastMNN	0.45	1.83
			Harmony	0.15	1.42
			PRECAST	1.82	0.81
			STADIA	1.92	1.97

References

A. Avalos-Pacheco, D. Rossell, and R. S. Savage. Heterogeneous large datasets integration using bayesian factor regression. *Bayesian Analysis*, 17(1):33–66, 2022.

K. Dong and S. Zhang. Deciphering spatial domains from spatially resolved transcriptomics with an adaptive graph attention auto-encoder. *Nature Communications*, 13(1):1–12, 2022.

M. Guilliams, J. Bonnardel, B. Haest, B. Vanderborght, C. Wagner, A. Remmerie, A. Bujko, L. Martens, T. Thoné, R. Browaeys, et al. Spatial proteogenomics reveals distinct and evolutionarily conserved hepatic macrophage niches. *Cell*, 185(2):379–396, 2022.

L. Haghverdi, A. T. Lun, M. D. Morgan, and J. C. Marioni. Batch effects in single-cell rna-sequencing data are corrected by matching mutual nearest neighbors. *Nature Biotechnology*, 36(5):421–427, 2018.

Y. Hao, S. Hao, E. Andersen-Nissen, W. M. Mauck, S. Zheng, A. Butler, M. J. Lee, A. J. Wilk, C. Darby, M. Zager, et al. Integrated analysis of multimodal single-cell data. *Cell*, 184(13): 3573–3587, 2021.

F. Hildebrandt, A. Andersson, S. Saarenpää, L. Larsson, N. Van Hul, S. Kanatani, J. Masek, E. Ellis, A. Barragan, A. Mollbrink, et al. Spatial transcriptomics to define transcriptional patterns of zonation and structural components in the mouse liver. *Nature Communications*, 12(1):7046, 2021.

L. Hubert and P. Arabie. Comparing partitions. *Journal of Classification*, 2:193–218, 1985.

A. L. Ji, A. J. Rubin, K. Thrane, S. Jiang, D. L. Reynolds, R. M. Meyers, M. G. Guo, B. M. George, A. Mollbrink, J. Bergenstråhle, et al. Multimodal analysis of composition and spatial architecture in human squamous cell carcinoma. *Cell*, 182(2):497–514, 2020.

I. Korsunsky, N. Millard, J. Fan, K. Slowikowski, F. Zhang, K. Wei, Y. Baglaenko, M. Brenner, P.-r. Loh, and S. Raychaudhuri. Fast, sensitive and accurate integration of single-cell data with harmony. *Nature Methods*, 16(12):1289–1296, 2019.

C. Liu and D. B. Rubin. Ml estimation of the t distribution using em and its extensions, ecm and ecme. *Statistica Sinica*, 5:19–39, 1995.

W. Liu, X. Liao, Z. Luo, Y. Yang, M. C. Lau, Y. Jiao, X. Shi, W. Zhai, H. Ji, J. Yeong, et al. Probabilistic embedding, clustering, and alignment for integrating spatial transcriptomics data with PRECAST. *Nature Communications*, 14(1):296, 2023.

Y. Long, K. S. Ang, M. Li, K. L. K. Chong, R. Sethi, C. Zhong, H. Xu, Z. Ong, K. Sachaphibulkij, A. Chen, et al. Spatially informed clustering, integration, and deconvolution of spatial transcriptomics with graphst. *Nature Communications*, 14(1):1155, 2023.

K. R. Maynard, L. Collado-Torres, L. M. Weber, C. Uytingco, B. K. Barry, S. R. Williams, J. L. Catallini, M. N. Tran, Z. Besich, M. Tippani, et al. Transcriptome-scale spatial gene expression in the human dorsolateral prefrontal cortex. *Nature Neuroscience*, 24(3):425–436, 2021.

C. E. Shannon. A mathematical theory of communication. *The Bell System Technical Journal*, 27(3):379–423, 1948.

R. R. Stickels, E. Murray, P. Kumar, J. Li, J. L. Marshall, D. J. Di Bella, P. Arlotta, E. Z. Macosko, and F. Chen. Highly sensitive spatial transcriptomics at near-cellular resolution with slide-seqv2. *Nature Biotechnology*, 39(3):313–319, 2021.

V. Svensson, S. A. Teichmann, and O. Stegle. SpatialDE: identification of spatially variable genes. *Nature Methods*, 15(5):343–346, 2018.

J. Zhu, S. Sun, and X. Zhou. SPARK-X: non-parametric modeling enables scalable and robust detection of spatial expression patterns for large spatial transcriptomic studies. *Genome Biology*, 22(1):1–25, 2021.
