# Supplementary figures and images for: Statistical batch-aware embedded integration, dimension reduction, and alignment for spatial transcriptomics
Source: Bioinformatics. 2024 Oct 14;40(10):btae611. doi: 10.1093/bioinformatics/btae611 (PMC11512591; doi:10.1093/bioinformatics/btae611)

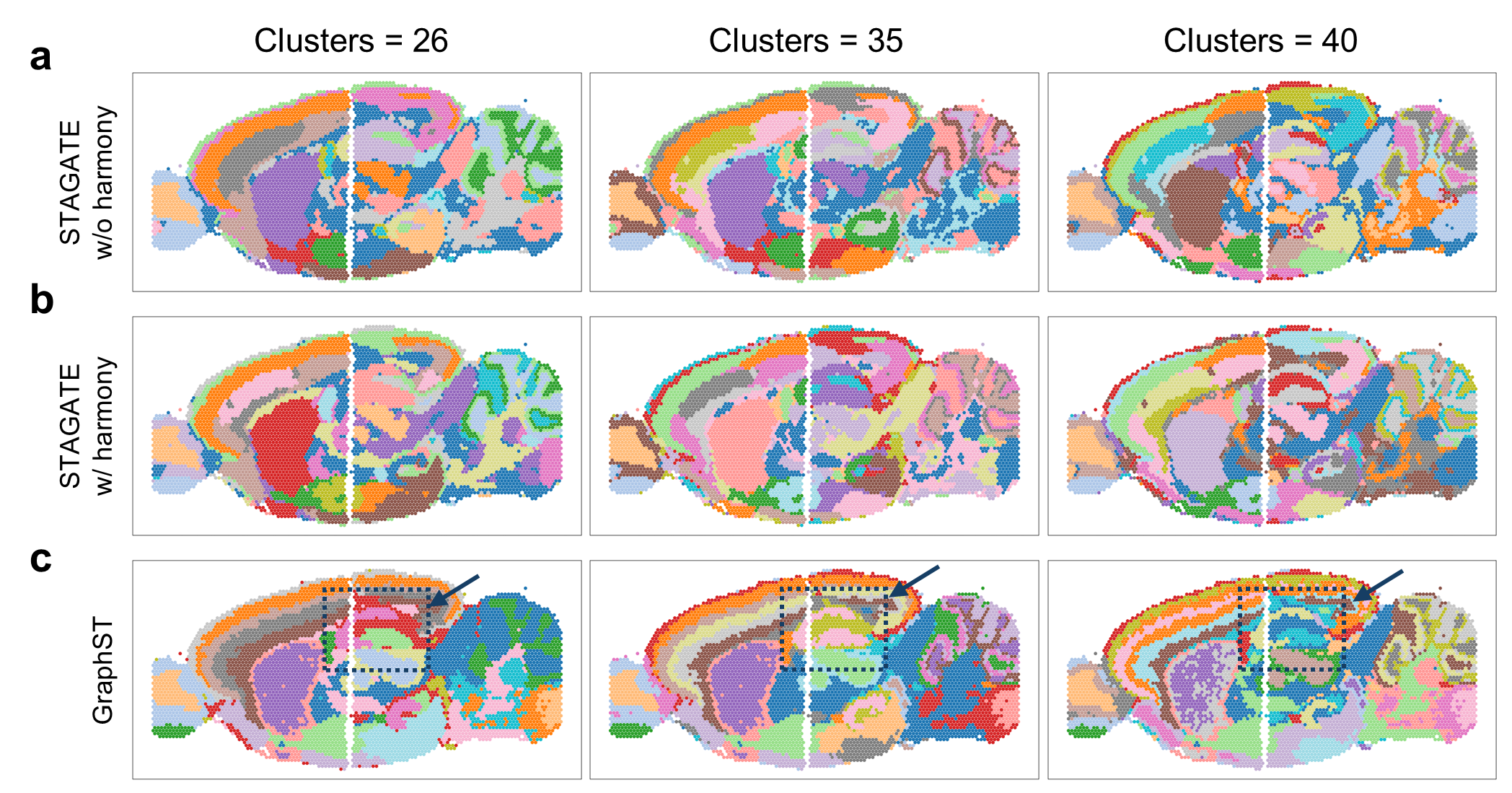

Supplement: btae611_Supplementary_Data [file btae611_supplementary_data.zip › FigS7.png]

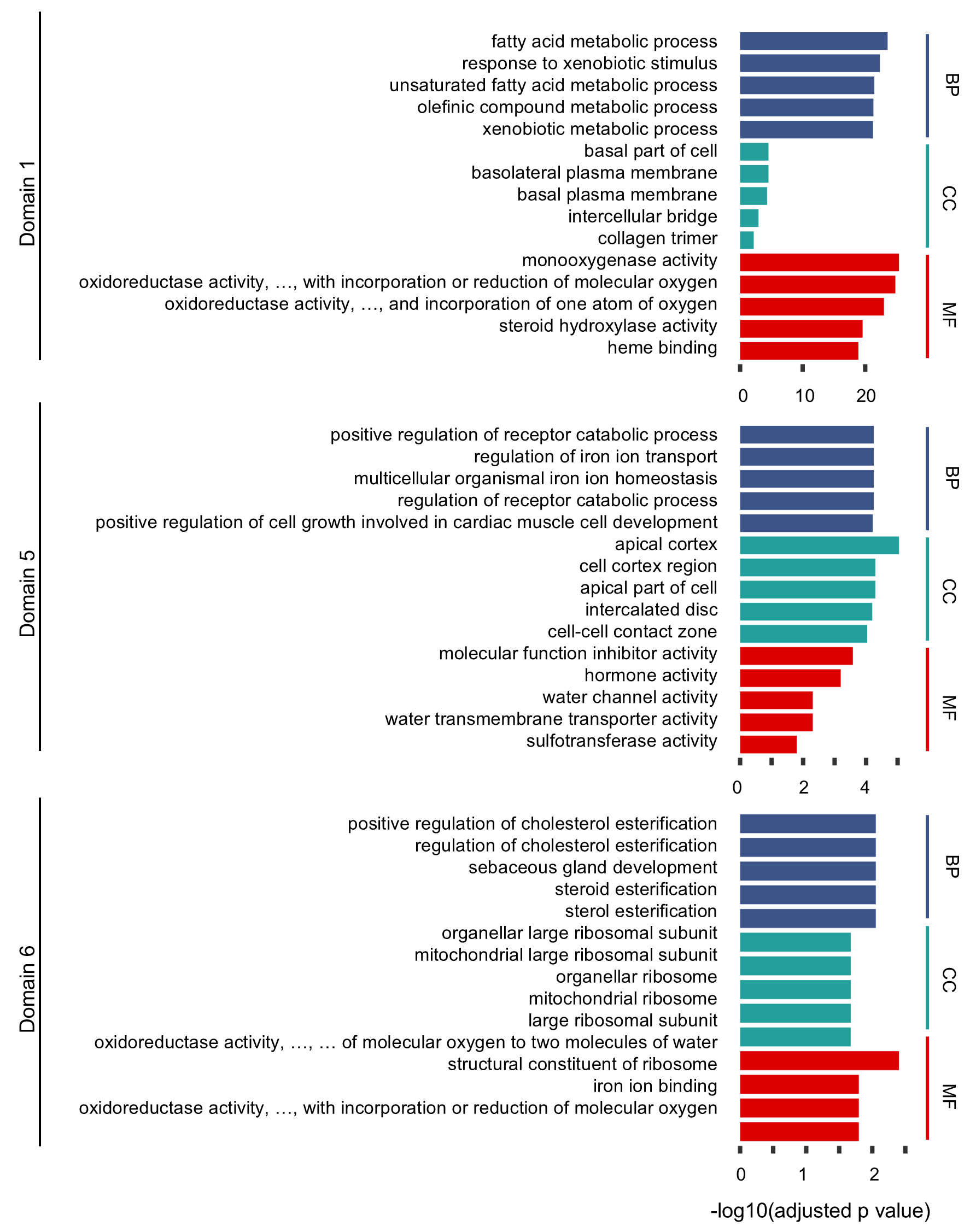

Supplement: btae611_Supplementary_Data [file btae611_supplementary_data.zip › figS3-2.png]

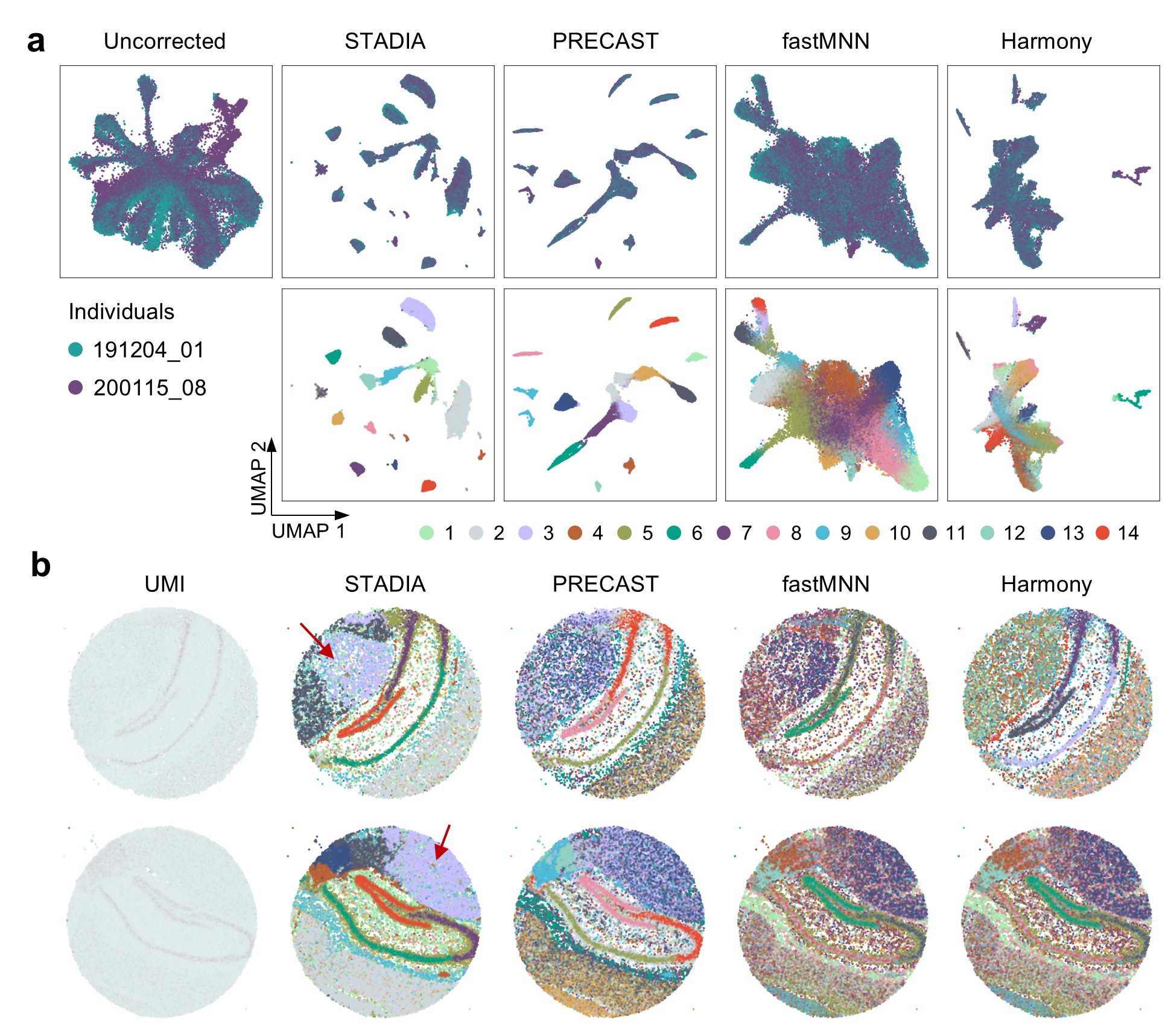

Supplement: btae611_Supplementary_Data [file btae611_supplementary_data.zip › figS4.png]

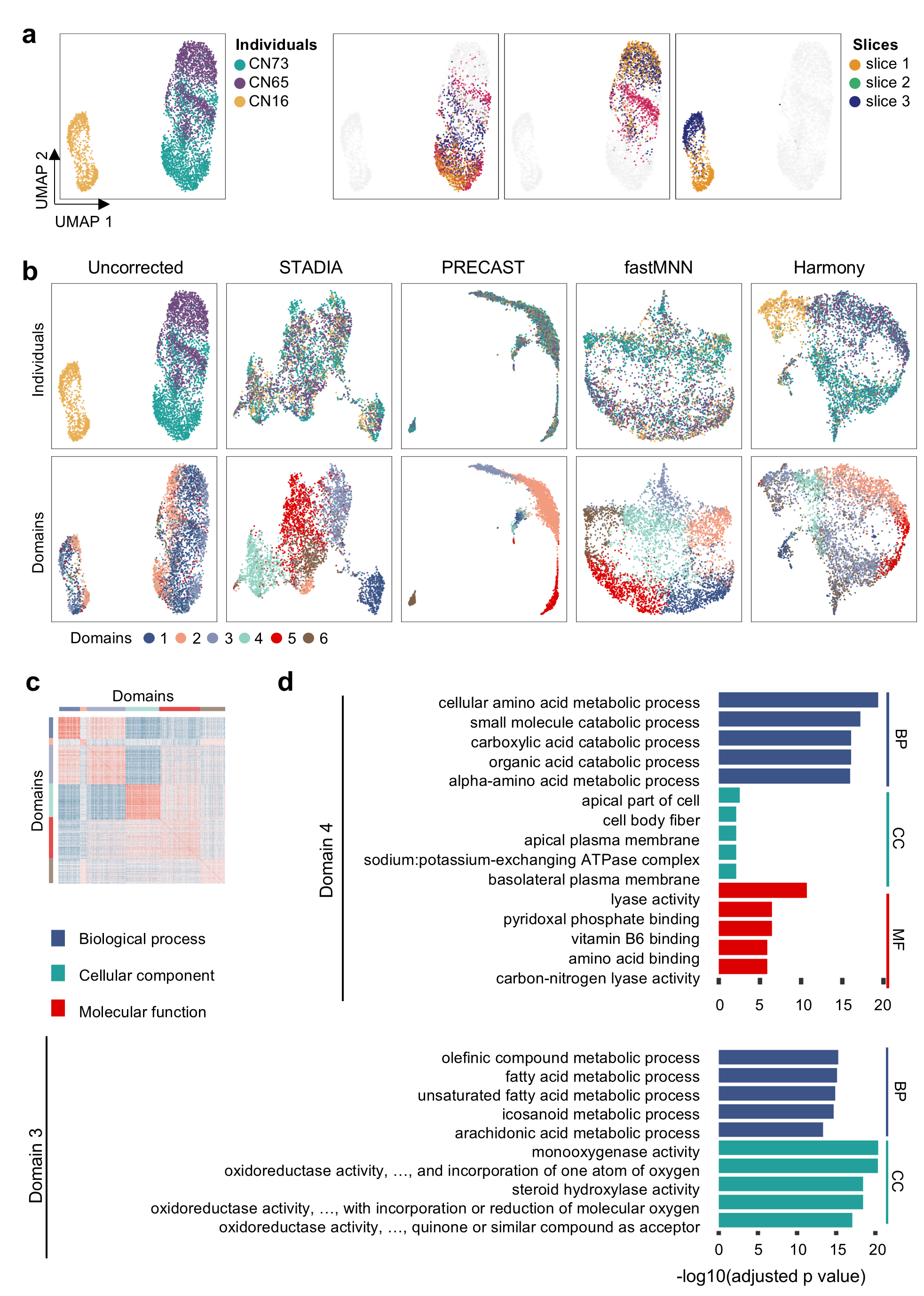

Supplement: btae611_Supplementary_Data [file btae611_supplementary_data.zip › figS3-1.png]

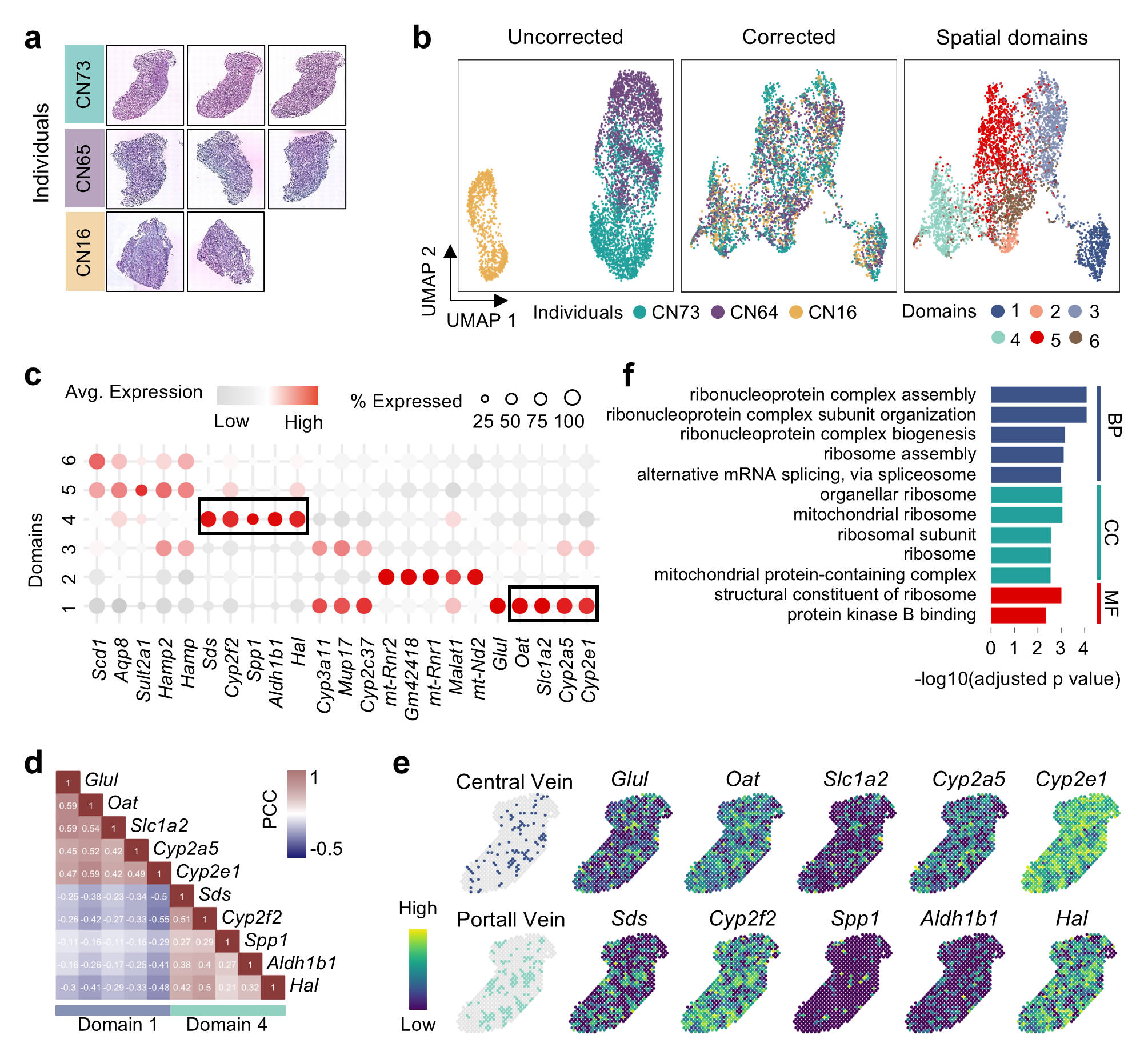

Supplement: btae611_Supplementary_Data [file btae611_supplementary_data.zip › figS5.png]

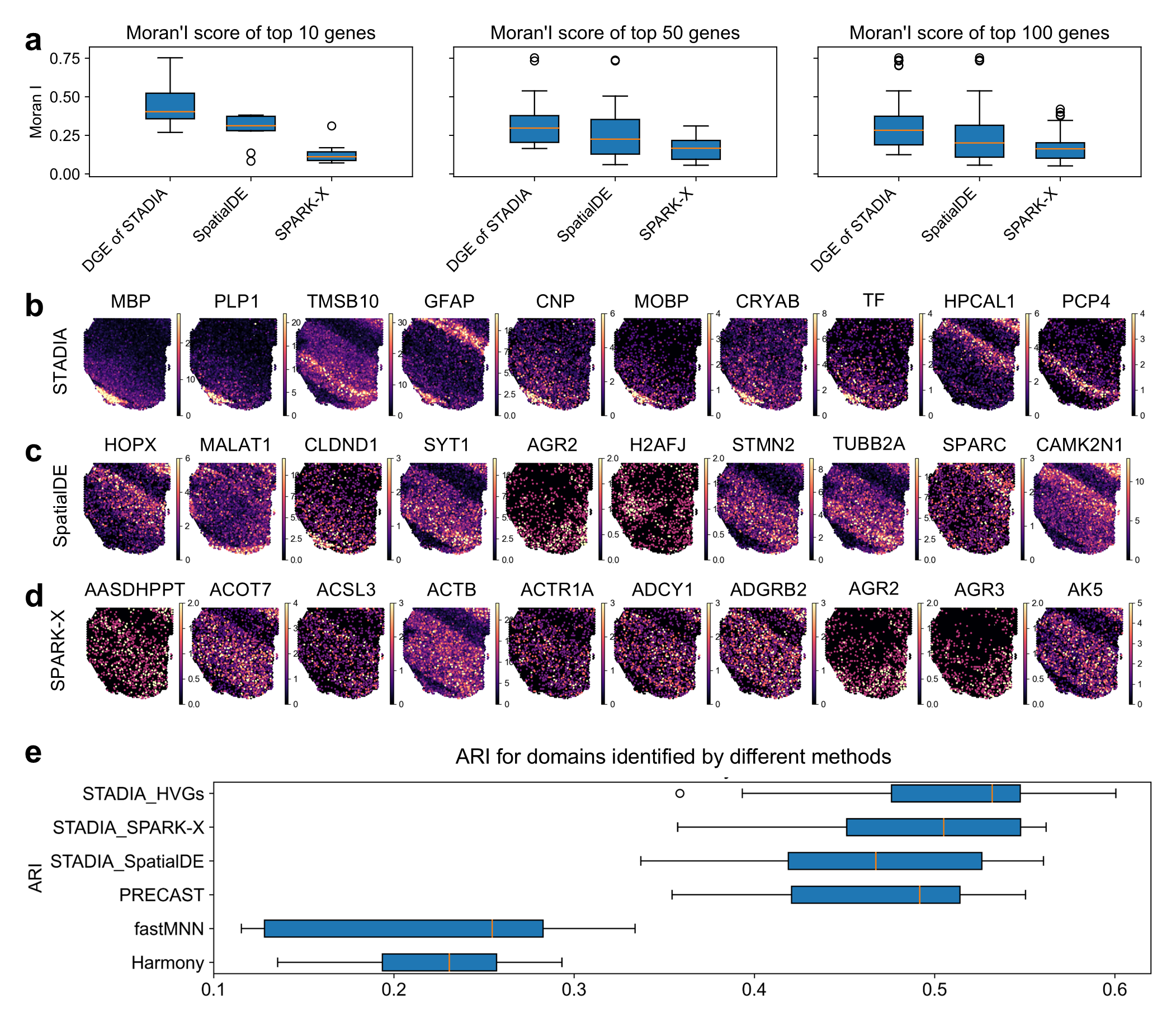

Supplement: btae611_Supplementary_Data [file btae611_supplementary_data.zip › FigS6.png]

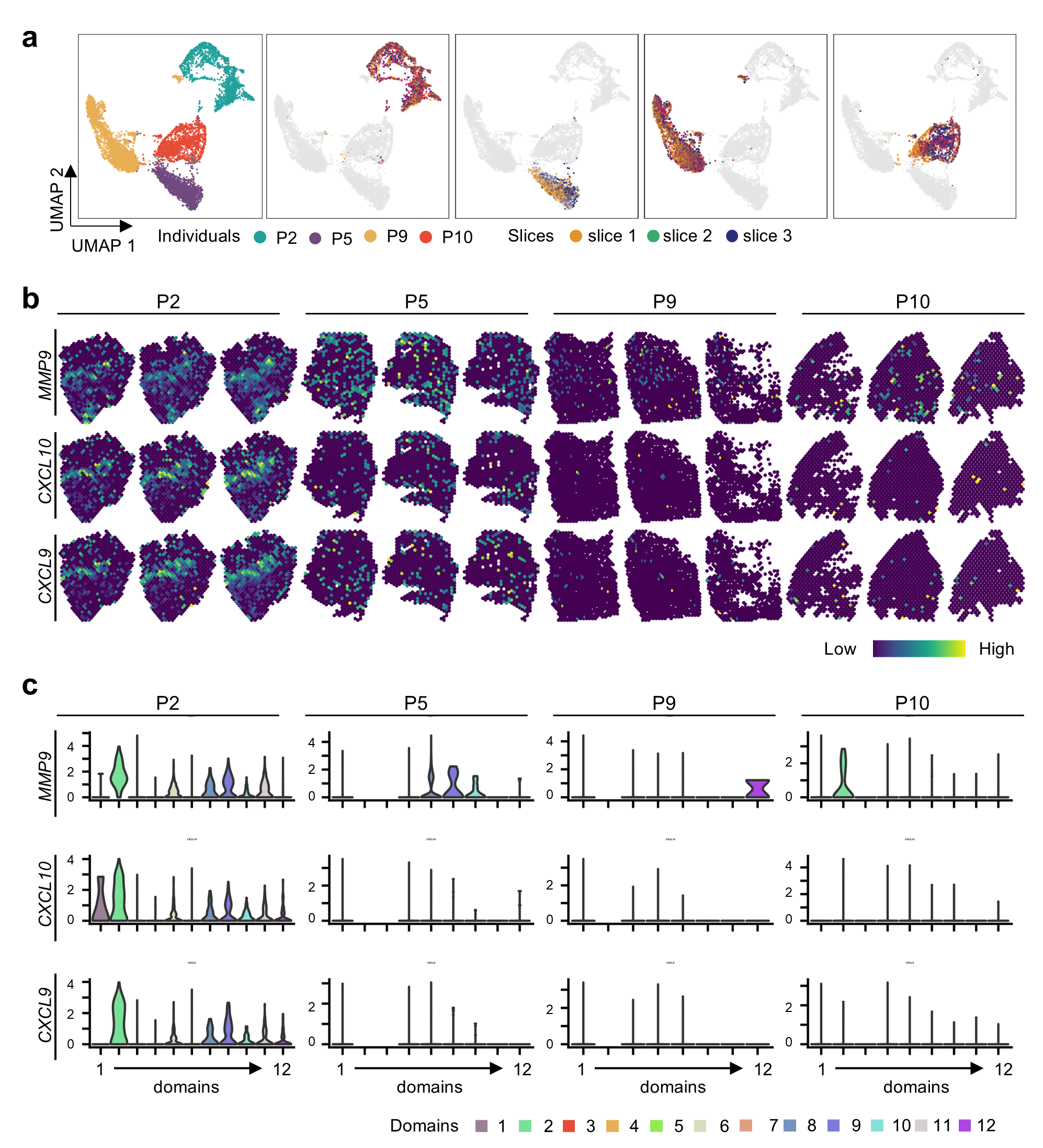

Supplement: btae611_Supplementary_Data [file btae611_supplementary_data.zip › figS2.png]

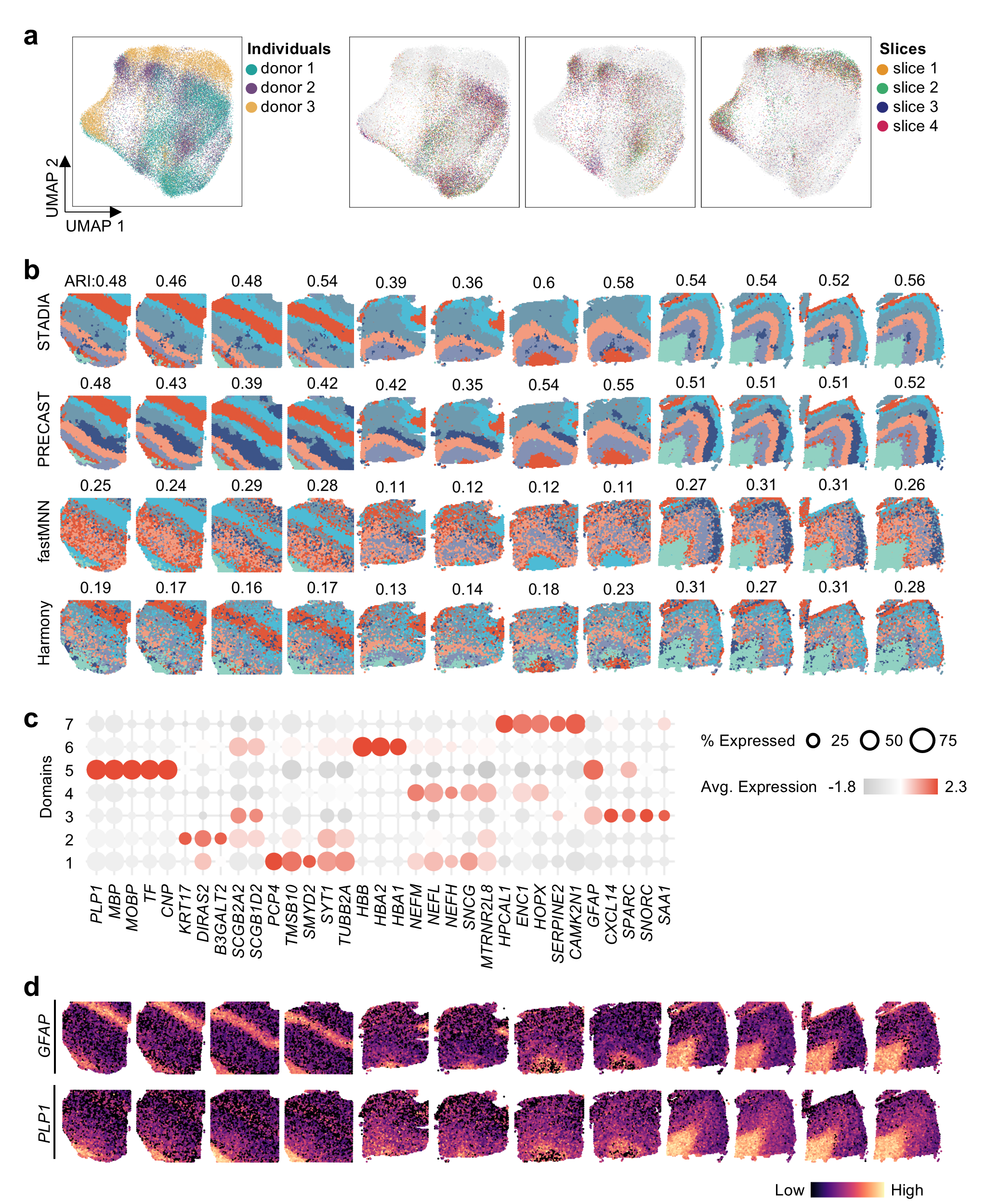

Supplement: btae611_Supplementary_Data [file btae611_supplementary_data.zip › figS1.png]

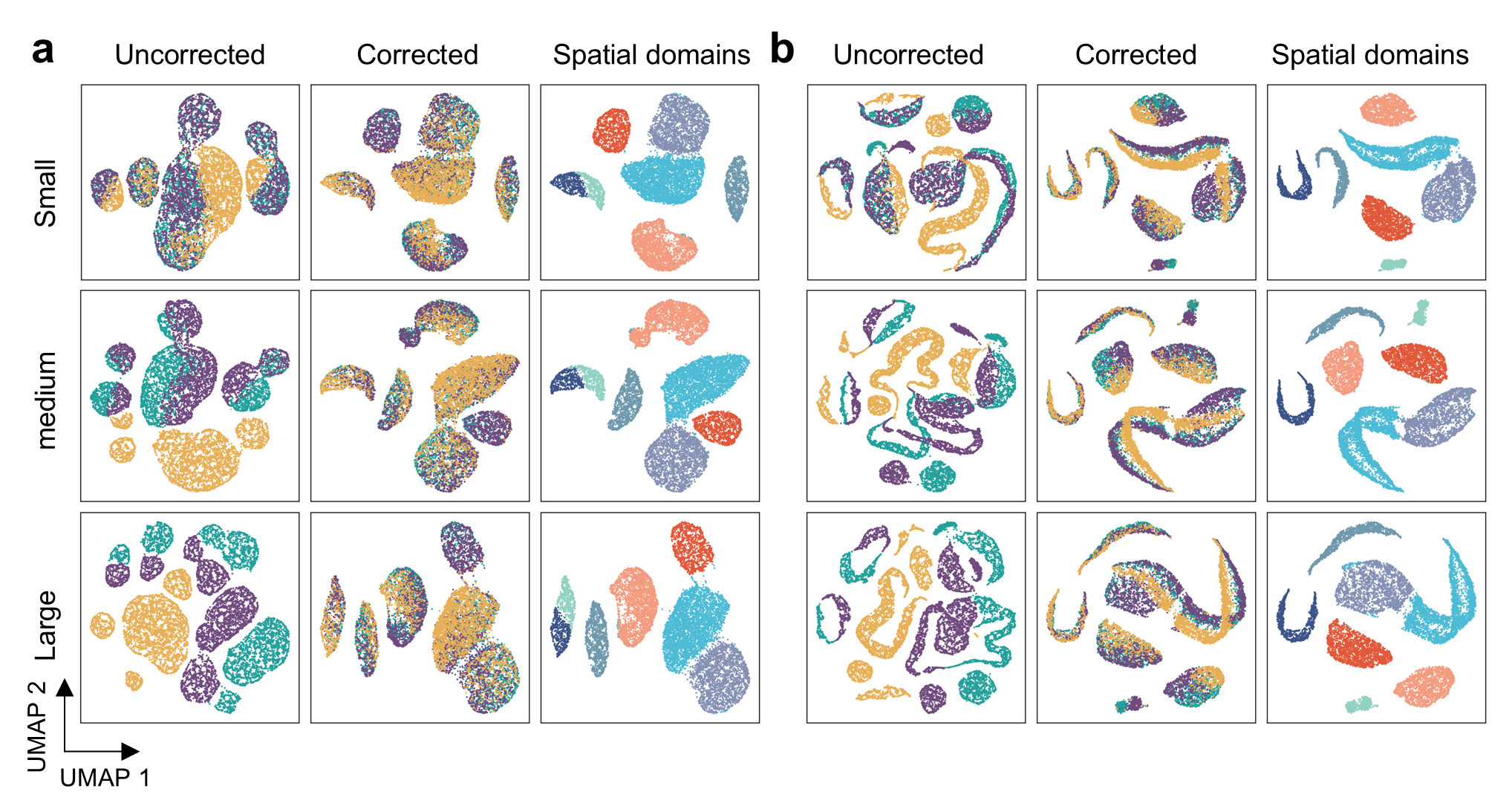

Supplement: btae611_Supplementary_Data [file btae611_supplementary_data.zip › FigS8.png]
